# Ploidy-stratified single cardiomyocyte transcriptomics map Zinc Finger E-Box Binding Homeobox 1 to underly cardiomyocyte proliferation before birth

**DOI:** 10.1007/s00395-023-00979-2

**Published:** 2023-03-02

**Authors:** Sara Thornby Bak, Eva Bang Harvald, Ditte Gry Ellman, Sabrina Bech Mathiesen, Ting Chen, Shu Fang, Kristian Skriver Andersen, Christina Dühring Fenger, Mark Burton, Mads Thomassen, Ditte Caroline Andersen

**Affiliations:** 1 Andersen Group, Department of Clinical Biochemistry, Odense University Hospital, Odense, Denmark; 2 Clinical Institute, University of Southern Denmark, Odense, Denmark; 3 Amplexa Genetics, Odense, Denmark; 4 Department of Clinical Genetics, Odense University Hospital, Odense, Denmark

**Keywords:** Heart development, Cardiomyocytes, Proliferation, Endoreplication, Zinc Finger E-Box Binding Homeobox 1 (Zeb1)

## Abstract

Whereas cardiomyocytes (CMs) in the fetal heart divide, postnatal CMs fail to undergo karyokinesis and/or cytokinesis and therefore become polyploid or binucleated, a key process in terminal CM differentiation. This switch from a diploid proliferative CM to a terminally differentiated polyploid CM remains an enigma and seems an obstacle for heart regeneration. Here, we set out to identify the transcriptional landscape of CMs around birth using single cell RNA sequencing (scRNA-seq) to predict transcription factors (TFs) involved in CM proliferation and terminal differentiation. To this end, we established an approach combining fluorescence activated cell sorting (FACS) with scRNA-seq of fixed CMs from developing (E16.5, P1, and P5) mouse hearts, and generated high-resolution single-cell transcriptomic maps of in vivo diploid and tetraploid CMs, increasing the CM resolution. We identified TF-networks regulating the G2/M phases of developing CMs around birth. ZEB1 (Zinc Finger E-Box Binding Homeobox 1), a hereto unknown TF in CM cell cycling, was found to regulate the highest number of cell cycle genes in cycling CMs at E16.5 but was downregulated around birth. CM ZEB1-knockdown reduced proliferation of E16.5 CMs, while ZEB1 overexpression at P0 after birth resulted in CM endoreplication. These data thus provide a ploidy stratified transcriptomic map of developing CMs and bring new insight to CM proliferation and endoreplication identifying ZEB1 as a key player in these processes.

**Supplementary Information:**

The online version contains supplementary material available at 10.1007/s00395-023-00979-2.

## Introduction

Cardiovascular diseases are among the leading causes of death worldwide [68], and one major explanation is the inability to regenerate the heart after myocardial infarction (MI), leaving the affected subjects with impaired cardiac function [52]. Whereas, the mammalian heart forms through cardiomyocyte (CM) proliferation during fetal development [58], CMs enter cell cycle arrest around birth [4]. As in general, CM proliferation requires the CM to go through the four cell cycle phases: G1, S, G2 and M [57]. Yet, around birth (P3 in mice) a portion of CMs exit the cell cycle and become quiescent in the G0 phase [28, 61, 71], whereas another CM fraction undergoes a final round of mitosis peaking at postnatal stage P5 (in mice), but indeed fails to complete karyo- and/or cytokinesis. This is often referred to as the G2/M challenge which results in polyploid and binucleated CMs [72, 79, 80], and is considered a hindrance for heart regeneration through CM proliferation [20, 32]. The mechanism for this switch is unclear, but has been associated with reduced expression of cyclins and cyclin dependent kinases (CDKs) in parallel with induced expression of several CDK inhibitors [29, 44], changes in miRNA expression, extracellular signaling pathways, centrosome integrity, telomere dysfunction, epigenetics as well as extracellular matrix compositions [51]. In the adult mouse and rat heart, ~ 80–95% [29] of CMs are binucleated while ~ 5% CMs are mononucleated [6]. In adult humans, controversies remain regarding the ratio of binucleated CMs compared to mononucleated, and it has been suggested that ~ 60% human CMs remains mononucleated [47], whereas the percentages of binucleated range between 12 and 75% [35]. Even so, a large portion of the mononucleated human CMs is polyploidy [47]. The terminally differentiated and polyploid CMs are often referred to as dormant with respect to cell division [32, 38], although recent evidence indeed contradicts this [36]. As with CM proliferation, current mechanistic insight to understanding polyploidy also remains deficient [32, 35]. More systematic approaches are therefore required to identify factors mediating CM cell cycle activity, and which on a longer term may be used to tackle the challenge of a non-regenerating heart.

Herein, we developed a new protocol combining fluorescence-activated cell sorting (FACS) of diploid and tetraploid murine CMs around birth with high-throughput single cell RNA sequencing (scRNA-seq) to uncover transcription factors (TFs) that regulate the G2/M CM cell cycle process.

## Methods

### Mice

C57BL/6 J mice were obtained from Taconic Europe, housed with a 12/12 h light/dark cycle, and fed ad libitum. For scRNA-seq, mice were plug bred, and litters for each of the three timepoints were obtained from different breeding pairs. Plug was checked in the morning and evening. For E16.5 primary cultures, mice were plug bred as well, whereas for primary P0 cultures continuous breeding was used. All animal experiments were approved by the Danish Council for Supervision with Experimental Animals (#2016–15-0201–00,941 and #2022–15-0201–01119).

### Preparation of CMs for scRNA-seq

For scRNA-seq, the left heart ventricle was dissected under a stereomicroscope at E16.5, P1, and P5 (*n* = 3 litters, each counting 4–8 pups), and enzymatically dissociated using the semiautomatic GentleMACS tissue dissociator system (MACS Miltenyi Biotec; Neonatal Heart Dissociation Kit 130–098-373) according to the manufacturer’s recommendations. Following viable cell counting (NC-200, ChemoMetec), dissociated cells were stained with a fixable viability stain (Fixable viability stain 570; BD Biosciences, 564,995) prior to fixing in methanol for 15 min followed by rehydration to reverse the RNA to its original state. During rehydration, the RNase inhibitor, RNasin Plus (Promega; N2615), was added to prevent RNA degradation and included in all subsequent steps. After rehydration, samples were stored at -80 °C until analysis. All reagents were high grade, RNase free and the environment was kept strictly RNase free to avoid degradation of the RNA. For comparison of fresh and fixed scRNA-seq profiles, mouse myoblasts (C2C12; ATCC, CRL-1772) were used and maintained as recommended.

### Fluorescence-activated cell sorting (FACS)

Fixed cardiac cells were stained for the CM marker MYH1 (Mouse IgG2b,k; 1:300; MF20-c; DSHB) and visualized by donkey anti-mouse IgG Alexa Fluor 488 (1:200; Invitrogen, A21202), whereas Hoechst 33342 (Sigma) was added 5 min before sorting (FACSAriaIII, BD Biosciences). Prior to FACS, cells were filtered (Falcon, 352235) to avoid cell clumps. Strict RNase free conditions as described above including new tubing were prioritized throughout the procedure. Analysis and sorting gating strategy (Supplementary Fig. 1c) included hierarchical gating using the FACSDiva software v8.0.1 (BD Biosciences) based on FSC/SSC, viability Alexa 570, and MYH1-Alexa 488, and Hoechst 33342. For each developmental stage (E16.5, P1, P5), three independent sortings (*n* = 3, each consisting of cells from one litter) were performed after carefully checking and validating the FACS setup using FMO controls (Supplementary Fig. 1c). Prior to scRNA-seq analysis CM purity, nuclei number, and cell clumping of sorted cells were assessed using immunofluorescence microscopy whereas the RNA integrity number (RIN) was determined using an Agilent 2100 Bioanalyzer (Agilent Technologies) combined with the Agilent RNA 6000 Nano Kit (Agilent Technologies) [56]. Sorted cells were stored at -80 °C until scRNA-seq.

### ScRNA-seq

For scRNA-seq, cells originating from three independent FACS were pooled (14–20 pups/sample) to account for biological diversity in the scRNA-seq analysis. Single Cell 3’ RNA-Seq libraries were prepared using Chromium Single Cell 3′ Reagent Kits v2 (10 × Genomics) according to the user guide. In brief, cellular suspensions of approx. 1200 cells/µl were mixed with master mix reagents and loaded on a Single Cell A Chip (10 × Genomics) together with Single Cell 3’ Gel Beads (10 × Genomics) and partitioning oil to generate single cell gel beads-in-emulsion (GEMs). The GEM generation took place in a Chromium Controller (10 × Genomics). Single cell reverse transcription was performed in a standard thermal cycler, and the GEMs were subsequently broken using Recovery Agent (10 × Genomics). The resulting cDNA was cleaned up with DynaBeads MyOne Silane Beads (Thermo Fisher Scientific) and SPRIselect Reagent Beads (Beckman Coulter), and then amplified by PCR using Single Cell 3′ Reagent Kit v2 (No. of cycles: 8). After another cDNA clean-up with SPRIselect Beads, the fragment sizes and concentrations were measured using QIAxcel DNA High Resolution Kit (1200) (Qiagen) and Qubit dsDNA HS Assay Kit (Thermo Fisher Scientific), respectively. Enzymatic fragmentation, end-repair, and A-tailing were performed in one-step using the Single Cell 3′ Reagent Kit v2, and fragments of approx. 200 bp were selected by double sided-size selection using SPRIselect Beads. NGS libraries were then constructed by adapter ligation and PCR mediated sample indexing (No. of cycles: 13). After a final double-sided size selection, the NGS library concentrations were measured using Qubit dsDNA Assay Kit. Libraries were sequenced on the Illumina NextSeq 500 platform using NextSeq 500/550, high output Reagent Cartridge V2, Illumina Kit (Read 1 = 26 cycles, i7 Index = 8 cycles, Read 2 = 130 cycles), and the second analysis was performed on a Illumina NovaSeq 6000.

### ScRNA-seq data analysis

*Read alignment and construction of gene expression matrix* Base calls were converted to FASTQ format and demultiplexed using the cellranger mkfastq function embedded in the 10 × Genomics cellranger software package using default settings (https://support.10xgenomics.com/single-cell-gene-expression/software/overview/welcome). Single cell gene counts matrices were generated using the cellranger count command. During this step, FASTQ files generated by the cellranger mkfastq step, were aligned to the *Mus musculus* genome (mm10/GRCm38) using the splice-aware aligner STAR [12]. Subsequently, STAR used the *Mus musculus* transcriptome reference (GRCm38.84) to segregate the mapped reads into exonic, intronic and intergenic regions and for assessment of how confidently the reads have been mapped to these regions. Only non-duplicated reads which were confidently mapped to the transcriptome, and which had barcodes and unique molecular identifiers (UMIs) were used for UMI counting. The expression matrices were generated by counting the number of strand-specific UMI for each cell mapping to either the exonic or intronic regions of each gene.

*Clustering and UMAP visualization* Using the R package Seurat[62] v 2.3.0 and 3.1.5 dimensionality reduction by principal component analysis (PCA) was performed; subsequently the PCA data analysis was used as input for visualization by Uniform Manifold Approximation and Projection (UMAP) clustering [69, 70]. Cell clustering by expression pattern was performed by first calculating the k-nearest neighbors and constructing the shared nearest neighbor (SNN) and next optimizing the modularity function to determine clusters.

*Clustering and Heatmaps* The Seurat v 2.3.0 and 3.1.5 [7, 62] were used for cluster visualization by UMAP and for differential gene expression of marker genes between clusters.

For each of the samples, a Seurat object was created, and the cells filtered based on whether they expressed a combination of the CM-specific markers *Tnni3, Tnnt2, Actc1,* and *Tnnc1*. Each sample was then log-normalized, variable features were identified using “vst” as selection method and 2000 nfeatures, and the data was scaled using nCount_RNA for vars.to.regress. PCA was run and based on jackstraw- and PC elbow plots the optimal number of dimensions was determined (range 9–14). Moreover, when applied, all samples were merged, integrated using FindIntegrationAnchors and IntegrateData, filtered, normalized, and scaled as described above with generated UMAP plots depicting cell cycle phases, clusters, and original data affiliation as for each individual sample.

*Visualization, clustering, and cell cycle analysis* UMAP plots and clusters were generated as described above using PCA as reduction type and resolution = 0.6; based on the top 30 marker genes for each cluster. Subsequent GO term enrichment was evaluated using clusterProfiler::enrichGO [75, 78] and the “org.Mm.eg.db” library with ont = “BP”, pAdjustMethod = “BH”, and cutoff values = 0.01. Features witg avg_log2FC > 0.5 were used, where each cluster was named according to biological identity. Finally, each dataset was split into three groups (G1-, S-, or G2/M-phase) based on the expression of cell cycle markers [67] and each cell was assigned with a cell cycle score using Seurat::CellCycleScoring.

*Analysis across developmental stages* After merging and integration as described above, two clusters of cell cycle active E16.5 and P5 cells, respectively, were subtracted from the data and compared using FindMarkers. The resulting list of features was used for generating cnetplot and TF analysis. Mouse single site analysis was used for TFs (oPOSSUM version 3.0) [25, 26, 33], all genes in current dataset as background, all vertebrate profiles with a minimum specificity of 8 bits, conservation cutoff 0.40, matrix score threshold 85%, up/downstream sequence 5000/5000). In addition, oPOSSUM results were supported by GSEA using the Molecular Signature Database [41, 42, 63] and transcription factor targets (TFT).

*Trajectory analysis* Data were prepared in Seurat (filtered and cell cycle assigned; since UMI data were used, normalization was avoided in agreement with recommendations by the Monocle platform) in merged pools of either 2n-, 4n-, or all samples, respectively. Subsequently, phenotype data and feature data were extracted from the Seurat object and converted to a Monocle CellDataSet (CDS) object. Next, dispersion estimates for count dataset were obtained using monocle::estimateDispersions and cells were sorted according to num_genes_expressed (500 < num_genes_expressed < 3000). A set of ordering genes was isolated using differentialGeneTest and used to order the CDS by the monocle::setOderingFilter. Next, the dimensions were reduced and cells were ordered along the trajectory using monocle::reduceDimension and monocle::orderCells, respectively. The trajectory was plotted depicting original identity, cell cycle phase, and pseudotime state. The monocle::BEAM function was utilized in each branch point of the trajectory plots to evaluate branch point dependent gene expression.

### Plasmids and AAV9 packaging

To determine the most efficient AAV serotype for CM transduction, pilot studies with both AAV6 and AAV9 transduction were performed, as these serotypes have previously shown efficient in CM transduction [53]. In our study design we found the AAV9 serotype to be much more efficient in transducing CMs, as compared to the AAV6 serotype (data not shown).

*Generation of plasmids* Plasmids harboring the genes of interest were purchased from Origene (Mouse Tagged ORF Clones; Supplementary Table 1), except for *Nfya*, which were synthesized by GeneArt (Thermo Fisher; Supplementary Table 1). The AAV backbone transfer vector was derived through modifications of the plasmid pAAV-EF1a-mCherry-IRES-Cre (a gift from Karl Deisseroth; Addgene plasmid # 55,632; http://n2t.net/addgene:55632; RRID:Addgene_55632) [15], allowing simultaneous transcription of mCherry and the gene of interest through the internal ribosome entry site (IRES). Thus, due to the IRES site, transcription of the gene of interest correlates to the level of mCherry. To unify the process of gene insertions, the restriction sites *SgfI* and *MluI* were inserted into the plasmid. Briefly, the already existing *MluI* restriction site was removed by introducing a point mutation in the plasmid by PCR amplification using the following primers: Forward: CGCACGGGTAAGCTTTGCAAAGATGGATAAAGTTTTAAACAGAGAGGA and Reverse: AAGCTTACCCGTGCGGCCGCAGGAACCCCTAGTGAT. The Cre site was then removed and the *SgfI* and *MluI* restriction sites were hereafter inserted by PCR amplification using the primers Forward: TCTGGTGCGATCGCCTAGACGCGTTAGATTCGATATCAAGCTTATCGATAATCAACCTCT and Reverse: CTAGGCGATCGCACCAGAACCACCATTATCATCGTGTTTTTCAAAGGAAAACCACGTCCC. Finally, a truncated chicken cardiac Troponin T promoter [53] (cTnT promoter; synthesized by GeneArt (Thermo Fisher); the DNA sequence was kindly provided by Professor Brent A. French, University of Virginia, USA) was inserted for CM specificity by PCR amplification in two steps: first, plasmid pAAV-EF1a-mCherry-IRES was PCR amplified by primers (Forward: GGAATTCCATATGGGTACCGGATCCGTGAGC and Reverse: GCTCTAGAAATTCCCACTCCTTTCAAGACCTAG) containing the *XbaI* and *NdeI* restriction sites to excise the EF1a promoter. Secondly, the cTnT promoter was inserted between the two restriction sites (Forward: GCTCTAGAGCAGTCTG and Reverse: GGAATTCCATATGAGGTC). The resulting pAcTnT-mCherry-IRES plasmid was then sequenced (Eurofins Genomics, Ebersberg, Germany) for validation (Data not shown). Genes (Origene plasmids and *Nfya*) were inserted into the pAcTnT-mCherry-IRES plasmid between the *SgfI* and *MluI* restriction sites. Since the *SgfI* restriction site was already included in the *Egr1* sequence, *Egr1* was amplified by the following primers Forward: AATGGTGGTTCTGGTGCGATCGCATGGCAGCGGCCAAG and Reverse: TTGATATCGAATCTAACGCGTGCAAATTTCAATTGTC. Next the *Egr1*sequence was added to pAcTnT-mCherry-IRES by NEBuilder® HiFi DNA Assembly Master Mix (NEB). Proper gene insertions were validated by enzymatic digestion at the respective restriction sites and size determined by gel electrophoresis (Data not shown).

For plasmid packaging in an AAV9 serotype capsid we used the Rep/Cap plasmid, pAAV2/9n, a gift from James M. Wilson (Addgene plasmid # 112,865; http://n2t.net/addgene:112865; RRID:Addgene_112865) and the helper plasmid pHelper (a kind gift to our collaborator Per Svenningsen, University of Southern Denmark, from Ben Deverman, Caltech, Pasadena, USA).

*Virus generation* Large-scale AAV generation for in vitro use was performed in HEK293T cells (ATCC; CRL-3216) by co-transfection with pAcTnT-mCherry-IRES (empty vector) or pAcTnT-mCherry-IRES harboring the gene of interest, pAAV2/9n and pHelper. Transfection efficiency was addressed by mCherry visualization using immunofluorescence microscopy. Five days after transfection, recombinant AAV was isolated by PEG 8000 precipitation and purified by iodixanol gradient ultracentrifugation followed by centrifugation through an Amicon Ultra Centrifugal filter (50 K). Recombinant AAV yields were determined by quantitative real-time PCR (qRT-PCR) through a titration of pAcTnT-mCherry-IRES plasmid using the primers Forward: AGTGTTGCATTCCTCTCTGG and Reverse: AGCGCATGAACTCCTTGAT.

Adenoviral constructs were generated by Vector Biolabs (PA, USA) using Adenoviral Human Type 5 (dE1/E3) as backbone. For ZEB1 knockdown experiments, a U6 promoter was driving ZEB1 short-hairpin RNA (shRNA) expression of the sequence 5´CCGGATAGAGGCTACAAGCGCTTTA-CTCGAG-TAAAGCGCTTGTAGCCTCTA-TTTTTTG-3´ and a targeting sequence of ATAGAGGCTACAAGCGCTTTA. An eGFP reporter was expressed under a separate CMV promoter. Ad-GFP-U6-scrmb-shRNA (cat. no. 1122N) containing a scrambled shRNA and an eGFP reporter was used as control. For ZEB1 overexpression experiments, the backbone vector contained a CMV promoter to drive expression of the gene of interest. Ad-GFP-Zeb1 was generated using mouse cDNA (GenBank: BC139768.1) and eGFP, and ZEB1 were expressed under separate CMV promoters. Ad-GFP (cat.no. 1060) was used as empty control.

### E16.5 CM cultures and Zeb1 knockdown

On embryonic day 16.5 (E16.5), the pregnant mice were sacrificed by cervical dislocation and the hearts from the pups were quickly removed and placed in a cardioplegic buffer (MIB; 1.2 mM KH_2_PO_4_ (pH 7.4); 0.25 g/l Na_2_CO_3;_ 6.44 g/l NaCl; 2.6 mM KCl; 1.2 mM Mg_2_SO_4_; 11 mM glucose) supplemented with 1% Bovine Serum Albumin (BSA; MIB/1%BSA). The heart ventricles were dissected under a stereomicroscope before enzymatically dissociation into a single cell suspension using the semiautomatic GentleMACS tissue dissociator system as described by the manufacturer. Dissociated cells were counted (NC-200; ChemoMetec), plated on extracellular matrix (ECM) at a density of approx. 118,500 cells/cm^2^_,_ and cultured in growth medium (79.5% DMEM (supplemented with 1% PenStrep (PS)), 19.5% Medium 199 (supplemented with 1% PS), and 1% newborn calf serum). After 24 h, the number of cells were counted in some wells to calculate the amount of virus required. Optimal MOI was determined from titrating the virus and quantifying transduction efficiency as well as observing for immediate cytotoxicity.

After 24 h of culturing, cells were transduced with 10 MOI of either Ad-GFP-shRNA or Ad-GFP-shRNA-Zeb1. In addition, 10 µM of 5-ethynyl-2′-deoxyuridine (EdU) was added to assess for cell cycle activity. The medium, with or without EdU, was replenished every 24 h, and experiments were terminated as indicated at 96 h after transduction for analysis.

### Neonatal CM cultures and viral transduction

Neonatal (P0) mouse pups from each litter were sacrificed by decapitation, whereafter the hearts were quickly removed, and the ventricles dissected under a stereomicroscope. Dissected ventricles were pooled in a tube with MIB/1%BSA before enzymatic dissociation into a single cell suspension using the semiautomatic GentleMACS tissue dissociator system as described by the manufacturer. Dissociated cells were resuspended in growth medium and the number of cells were counted (NC-200; ChemoMetec). Cells were seeded in 12-well plates pre-coated with ECM at a density of approx. 236,500 cells/cm^2^ and placed in an incubator (37 °C, 5% CO_2_) or approx. 88,235 cells/cm^2^ on 4-well chamber slides (cat.no. 154917, Lab-Tek™ II) for confocal microscopy.

Before deciding which concentration of virus to use, both for AAV9 and adenovirus transduction, titration tests were performed and the concentrations resulting in the most efficient transduction without causing cytotoxicity were used. After 24 h the number of cells in each experiment were estimated (NC-200; ChemoMetec), and cell cultures were transduced with either 750,000 viral genomes (vg)/cell of the desired AAV9 or 50 MOI of adenovirus. For AAV9 experiments, six, 24, and 48 h after viral transduction, medium was refreshed with medium containing 10 µM EdU. For adenovirus, EdU was added together with the virus and replenished every 24 h. All cells for qRT-PCR were replenished with medium without EdU. Cells were either fixed in 2.5% Neutral Buffered Formalin (NBF) diluted in HBSS/5%FBS/1%PS for flow cytometry analysis 72 h post transduction (see below), fixed in the wells in 10% NFB or 4% Paraformaldehyde (PFA), or the RNA was isolated for qRT-PCR 48 h post transduction for adenovirus or 72 h post transduction for AAV9 (see below). Transduction efficiency was addressed by immunofluorescence microscopy for mCherry during culture, and by qRT-PCR (mCherry and/or gene of interest) and flow cytometry for mCherry or GFP. Furthermore, we consistently observed lower levels of GFP with the Ad-GFP-Zeb1 compared to Ad-GFP suggesting correlation between GFP and ZEB1 expression.

### Flow cytometry

Fixed cells were permeabilized with phosphate buffered saline (PBS) containing 1% BSA and 0.1% Triton X-100 (TX100) and stained with primary antibodies in different combinations (mouse anti-MYH1, 1:300, MF20-c, DSHB; rat anti-mCherry, 1:500, M11217, Thermo Fisher; and rabbit anti-GFP, 1:500, ab290, Abcam) for 1 h in the dark on ice while shaking. After washing, cells were incubated with EdU Click-it reaction cocktail according to the manufacturer’s protocol (Invitrogen, C10419), and washed before incubation with secondary antibodies in different combinations (488-donkey anti-mouse, 1:200, A21202, Invitrogen; 555-donkey anti-rat, 1:200, Ab150154, Abcam; 555-donkey anti-mouse, 1:200, A31570, Invitrogen; and 488-donkey anti-rabbit, 1:200, A21206, Invitrogen) for 30 min in the dark on ice while shaking. Three final washes were performed in PBS/1% BSA/0.1% TX100 and Hoechst 33342 was added 5 min before flow cytometry using the LSRII flow cytometer (BD Biosciences). Data was analyzed using the FACSDiva software v8.0.1, and initially gated according to the CM marker MYH1 and then sub-fractionated based on the antibody amplified mCherry or GFP signal. Cells positive or negative for a reporter (mCherry or GFP) were gated according to EdU incorporation to determine cell cycle activity. Ploidy was addressed in subpopulations by Hoechst 33342 using gates (2n, 4, and > 4n) defined by the entire CM population. Each analysis as indicated consisted of at least three to nine independent experiments designated n, each comprising cells from one litter. Within an experiment, 1–3 replicates (n*) were performed and used as an average of the *n* for further statistical analysis as indicated.

### RNA isolation, RNA integrity, and qRT-PCR

RNA was isolated from each sample and qRT-PCR was performed as described previously [3]. Briefly, the cells were lysed with TriReagent and the RNA was isolated using Polyacryl carrier, 1-Bromo-3-Chloro-Propane and 2-propanol. The RNA was rinsed using 75% ice cold ethanol. Finally, the RNA was dissolved in nuclease-free water and the RNA concentration was determined using a nano-drop. For qRT-PCR, cDNA was generated using the High-Capacity cDNA Reverse Transcriptase kit (Applied Biosystems; 4368814) according to the manufacturer’s recommendations. Each sample for qRT-PCR contained 2–4 ng cDNA (Supplementary Table 2) in a total volume of 10 µl and were analyzed in technical triplicates of qRT-PCR using a mixture of Power SYBRGreen PCR Master Mix (Applied Biosystems, 4367659) and appropriate forward and reverse primers (Supplementary Table 2). The qRT-PCR was run on a 7900HT Fast Real-time PCR system (Applied Biosystems) under the following conditions: holding for 10 min at 95 °C, hereafter 40 cycles consisting of 15 s of denaturation at 94 °C, 30 s of annealing at 57–60 °C (Supplementary Table 2) and 30 s of elongation at 72 °C. The obtained data was analyzed as previously described [3] by normalization to multiple stably expressed endogenous gene according to the qBase Plus 3.2 platform (Biogazelle).

### Injection of adenovirus in P0 pups

The litter was gently taken from their home cage. Pups were then anesthetized by induction of hypothermia before 7.60 × 10^14^ PFU (in a total volume of 20 µl, diluted in sterile PBS) of the desired adenovirus was injected into the superficial temporal vein. Pups were reheated and placed together with their littermates before the litter was gently put back into their home cage. Following the pups received a 50 µl subcutaneous injections of EdU (2.5 mg/ml) at P4 and P6 before they were sacrificed by decapitation at P8. Hearts were dissected and either dissociated for flow cytometry as described above for P0 pups, except cells were strained (100 µm nylon cell strainer, 352360) prior to NBF fixation, and each heart was processed individually, or prepared for paraffin embedding (see below).

### Immunohisto- and immunocyto-chemistry and microscopy

P8 hearts from virus injected pups were fixed in 10% NBF overnight, rinsed in PBS, dehydrated, and finally embedded in paraffin. Embedded specimens were then cut into 10 µm sections before mounting on glass slides and stored at 4 °C. Sections were deparaffinized and rehydrated before staining. Immunostainings were performed on NBF or PFA fixed cell cultures or paraffin embedded sections as previously described in Andersen et al. [2] with the following primary antibodies: rabbit anti-GFP (1:500, ab290, Abcam), mouse anti-MYH1 (1:300, MF20-c, DSHB), rabbit anti-ZEB1 (1:500, PA5-28,221, Thermo Fisher), rabbit anti-Mef-2c (1:500, 5030S, Cell Signaling Technology), mouse anti-actinin (1:200 A7811, Sigma), mouse anti-α-tubulin (1:250, 3873, Cell Signaling Technology), and 647-phalloidin (1:800, A30107, Thermo Fisher). Secondary antibodies used were: 488-donkey anti-rabbit (1:200, A21206, Invitrogen), 555-donkey anti-rabbit (1:200, A31570, Invitrogen), 647-donkey anti-mouse (1:200, A31571, Invitrogen). All sections were mounted with DAPI (Vectashield, Vector Lab., for Phalloidin and α-tubulin staining, Fluoroshield, Abcam was used). Microscopy was performed on a Leica DMI 4000 B microscope with a Leica CTR4000 illuminator and Leica DFC300FX/DFC 340 FX cameras, and confocal microscopy was performed on an Olympus FV1000MPE confocal laser scanning microscope equipped with an UPlanSApo 60x/1.20 water objective. During analysis, all camera settings and picture processing were applied equally to samples and controls.

### Statistics and reproducibility

All statistics were performed using the GraphPad Prism (v 9.0.0) software and the appropriate tests, number of independent experiments (*n*) and replicates (n*) are defined in the corresponding figure legends. We used the significance level α = 0.05 for identifying significant results marked by asterisks, yet have indicated throughout the exact *p*-value if between 0.05 and 0.1 to enable objective evaluation of trends. For scRNA-seq, each timepoint includes three biologically independent experiments each comprising mouse pups derived from three distinct litters. Cells were pooled just before library generation and scRNA-seq. ScRNA-seq and subsequent gene expression analysis and transcription factor binding site analysis was performed using two different sequencing platforms (NextSeq and NovaSeq) with similar results. All transduction experiments were successfully reproduced with at least three biologically independent experiments obtaining similar results, thereby confirming the design and robustness.

## Results

### FACS of fixed CMs combined with high-throughput scRNA-seq efficiently distinguishes diploid and tetraploid CMs during heart development

**Fig. 1:**
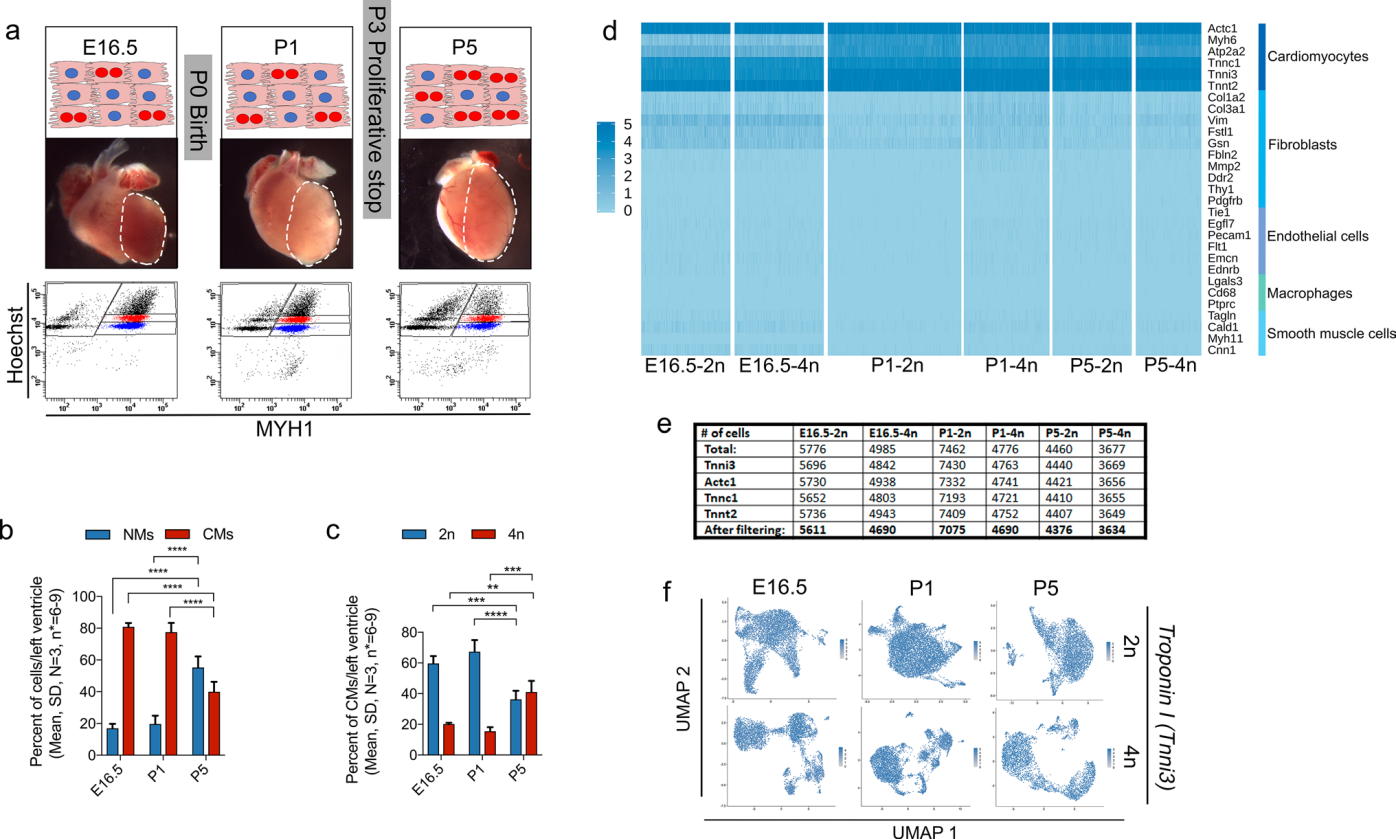
**High scRNA-seq CM resolution of embryonic and neonatal CMs a** Top: Schematic of the CM binucleation process during heart development (E16.5, P1, and P5). Middle: Images of mouse hearts used for scRNA-seq with the left ventricle being dissected and used (encircled by the dotted line). Bottom: FACS dot plots showing the CM marker MYH1-488/Hoechst-stained cells (*N* = 3 litters/age; 14–20 pups/age). **b–c** Quantification of percentage of **b** non-myocytes (NMs) and CMs, **c** and 2n- and 4n-CMs, at E16.5, P1, and P5 based on flow cytometric data and cell counts (mean, SD, *N* = 3, Statistical analysis included two-way ANOVA with Tukey’s post test; ****P ≤ 0.0001). **d** Heatmap visualizing differential expression of known marker genes for different cell types (CMs, fibroblasts, endothelial cells, macrophages, and smooth muscle cells) in scRNA-seq analyzed heart cells (2n- and 4n, E16.5, P1 and P5) prior to analytical CM-filtering. **e** Table displaying the number of cells prior to and after analytical CM filtering according to expression of the CM marker genes *Tnni3, Actc1, Tnnc1* and *Tnnt2* at E16.5, P1 and P5, and stratified by ploidy (2n and 4n). **f** UMAP-plots showing the expression of the CM marker *Tnni3* prior to filtering (blue: *Tnni3*^+^, grey: *Tnni3*^*−*^*)*

To address the mechanisms underlying CM proliferation in the G2/M phases, our focus was fairly strict on tetraploid CMs in the G2/M phases of the cell cycle (Fig. 1a). To that end, we developed an approach that combines FACS of fixed heart cells with scRNA-seq of the sorted CMs (Supplementary Fig. 1a), while preserving the RNA integrity (RIN; Supplementary Fig. 1b) to increase resolution specifically of CMs in the scRNA-seq analysis. Advantages of this method (Supplementary Fig. 1a) includes avoidance of the long-term susceptibility of CMs to tissue dissociation, improvement of logistics with the ability to store scRNA-seq samples obtained at different timepoints, and application of FACS to enrich CMs using intracellular CM markers such as myosin heavy chain 1 (MYH1) and DNA (Hoechst; Supplementary Fig. 1a–c). Based on a viability stain prior to fixation we excluded and reduced the number of dead cells (Supplementary Fig. 1c) and in general checked for clumps of cells (Supplementary Fig. 1e, f) prior to generating cDNA libraries for scRNA-seq. Overall, scRNA-seq data from fresh and fixed cells show a high degree of correlation (*R* = 0.98; *p* < 2.2 × 10^–16^, Supplementary Fig. 1d) confirming data consistency between the methods.

We next aimed to use this approach for identifying TFs specific for regulating the G2/M phases of CMs around birth. Accordingly, we isolated diploid (2n) and tetraploid (4n) CMs from the left ventricle of mice before (E16.5 and P1 samples) and after (P5 samples) (Fig. 1a) the known proliferative stop at P3 [61]. At E16.5, the left ventricle comprised of 80.8 ± 2.4% CMs and 17 ± 2.8% non-myocytes (NMs) (mean, SD; *n* = 14–20). This CMs/NMs ratio remained constant from E16.5 to P1 with 77.5 ± 5.8% CMs and 19.6 ± 5.3% NMs at P1, whereas the percentage of NMs as expected increased rapidly from P1 to P5 (55.3 ± 6.9% NMs) (Fig. 1b). No difference was observed in the percentage of diploid CMs (2n-CMs) between E16.5 (59.7 ± 4.9%) and P1 (67.4 ± 7.5%), but this percentage declined (36.2 ± 5.7%) at P5 (Fig. 1c). Likewise, the percentage of tetraploid CMs (4n-CMs) remained constant from E16.5 (20.2 ± 0.8%) to P1 (15.5 ± 2.7%), but increased to 41 ± 7.3% at P5 (Fig. 1c). Thus, as expected karyo-/cytokinesis failure in the G2/M phases of P5 CMs were apparent. By examining the expression of MYH1 and the number of visible nuclei in FACS sorted P5 CMs, we found that > 95% of the diploid and 50% of the tetraploid CMs were mononucleated, whereas 40% of the tetraploid CMs were binucleated (Supplementary Fig. 1e, f). The CM purity was > 95% and cell clumps were scarce (Supplementary Fig. 1e, f), the latter minimizing the appearance of false positive intermediates in the subsequent scRNA-seq analysis. High-throughput scRNA-seq with NextSeq (266,839,970 reads in total for 6 samples) on sorted 2n- and 4n CMs from E16.5, P1, and P5 revealed a total of 31,156 cells passing quality control filters with an average of 5,193 cells/sample and 1,135 genes in average per cell identified (Supplementary Fig. 1 g). The cells were sequenced at a median depth of 9,278 reads per cell with a median alignment rate of 74% per cell. Subsequent preparation of data for analysis was performed using the R package Seurat [62]. The total number of genes identified in all samples was 14,222 prior to any filtering. Expression of CM markers (Fig. 1d,e) confirmed a 96.85% CM purity of analyzed cells, yet, to exclude the few contaminants, we prefiltered the data by defining CMs as those with a combined expression of *Tnni3, Tnnt2, Tnnc1*, and *Actc1* (Fig. 1e,f). This resulted in an average of 5136 CMs per sample (Fig. 1e). After filtering, the remaining data for only CMs were log-normalized, variable genes were identified, and data were scaled according to the total detected number of molecules in each cell (nCount_RNA). Thus, our new approach combining FACS of fixed CMs with scRNA analysis enabled us to increase scRNA-seq resolution with high numbers of CMs stratified by ploidy.

### Gradual CM maturation and terminal differentiation is a dynamic process occurring already at E16.5, leaving P1 hearts almost devoid of proliferating CMs

**Fig. 2:**
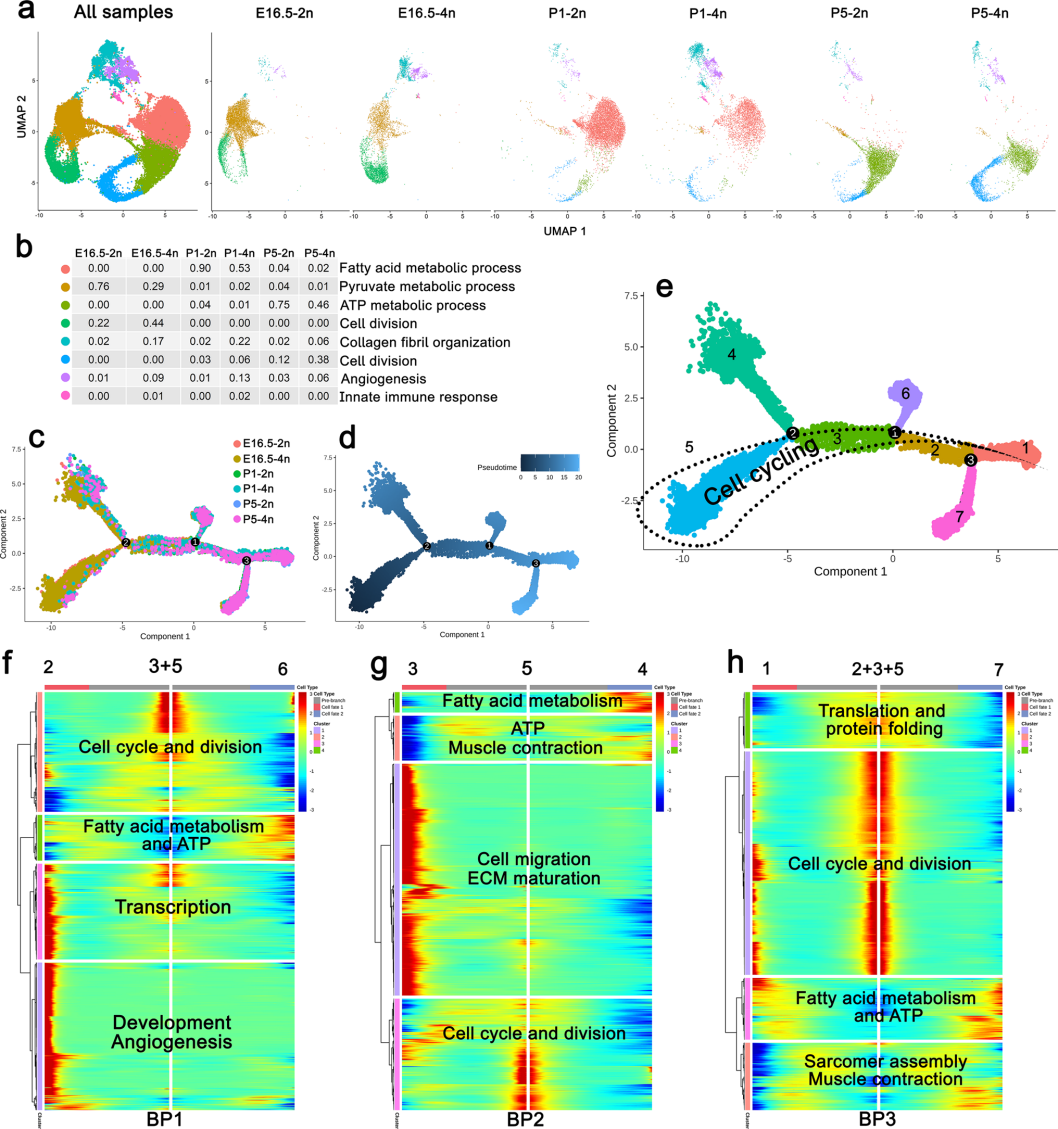
**CM cell cycle exit, and maturation are initiated before birth. a** UMAP clustering based on k-nearest neighbors of CMs with representative GO terms and ratio composing each cluster for each ploidy/developmental stage **b** with colors referring to different clusters. **c**, **d**, **e**, Trajectory analysis by Monocle displaying original identity of cells **c**, pseudotime **d**, and trajectory state including three branchpoints (BP); The overall expression pattern of genes related to cell cycling from BP heatmaps (below) is marked by a dotted line **e**. **f**, **g**, **h**, Heatmaps showing differentially expressed genes during CM development along pseudotime with dominating GO terms for each cluster in each BP indicated. Hierarchical clustering is based on Ward.D2 and unrelated to the clustering in (**a**, **b**). Above each heatmap, the states of the branches (according to (**e**)) are designated

To evaluate the biological activity of the sorted CMs, all scRNA-seq samples were integrated and CMs were clustered and visualized based on their gene expression profile, using Uniform Manifold Approximation and Projection (UMAP) plots combined with Gene Ontology (GO) term designation (Fig. 2a, b). In agreement with prior knowledge [13, 45], we observed that embryonic CMs switch from pyruvate metabolism to fatty acid metabolism soon after birth (Fig. 2a, b), validating the scRNA-seq design. Moreover, we identified two major “Cell division” clusters (Fig. 2a, b), one mainly composed of E16.5 CMs and one dominated by P5 CMs, both embracing mainly tetraploid CMs. Using trajectory analysis (Fig. 2c, d), we found an overall ordering of the CMs in pseudotime that corresponds to the relative developmental stage, with E16.5 CMs mainly represented early in pseudotime, after which they mature through the P1 stage and ending as P5 CMs (Fig. 2c, d and Supplementary Fig. 2a). Yet, a small fraction of E16.5 CMs, especially tetraploid CMs, did also align late in pseudotime, while some P1 and P5 CMs resided in the early branches according to pseudotime (Fig. 2c, d and Supplementary Fig. 2a). This may suggest that the process of CM maturation and likely cell cycle exit is dynamic and ongoing already at E16.5, whereas a small fraction of CMs at P5 may exhibit an immature phenotype similar to E16.5 CMs. This was further confirmed by branchpoint analysis (Fig. 2e–h, Supplementary Fig. 2b) showing that the two branches early in pseudotime (Fig. 2e, Supplementary Fig. 2b) primarily differ by metabolism and cell cycling where the earliest trajectory encompasses less differentiated CMs (Fig. 2g). Particularly, we observed a higher cell cycling activity for the lower trajectory of 4n-CMs (Supplementary Fig. 2b). The later bifurcations from the main trajectory all seem to exhibit lower cell cycle activity (Fig. 2e) but higher level of CM differentiation (“Shape and adhesion”, “ECM maturation”, “Fatty acid metabolism”, “Sarcomere assembly”, and “Muscle contraction”) as well as “Cell Migration” (Fig. 2c). Cell cycle activity was, however, regained for 4n-CMs at the tip of the lower trajectory (Supplementary Fig. 2b). Since the P5 cell cycle active CMs remain after the proliferation stop at P3 [61], and also exhibit mature CM characteristics, the P5 cell cycle activity most likely represent binucleation/polyploidization. Moreover, the data suggest that already before birth (E16.5) a small portion of CMs escape from cell division and starts maturing rendering P1 hearts almost devoid of CMs exhibiting cell division properties.

### The cell cycling machinery of cycling E16.5 tetraploid G2/M CMs is defined by a certain set of TFs different from that in P5 cycle active tetraploid G2/M CMs

**Fig. 3:**
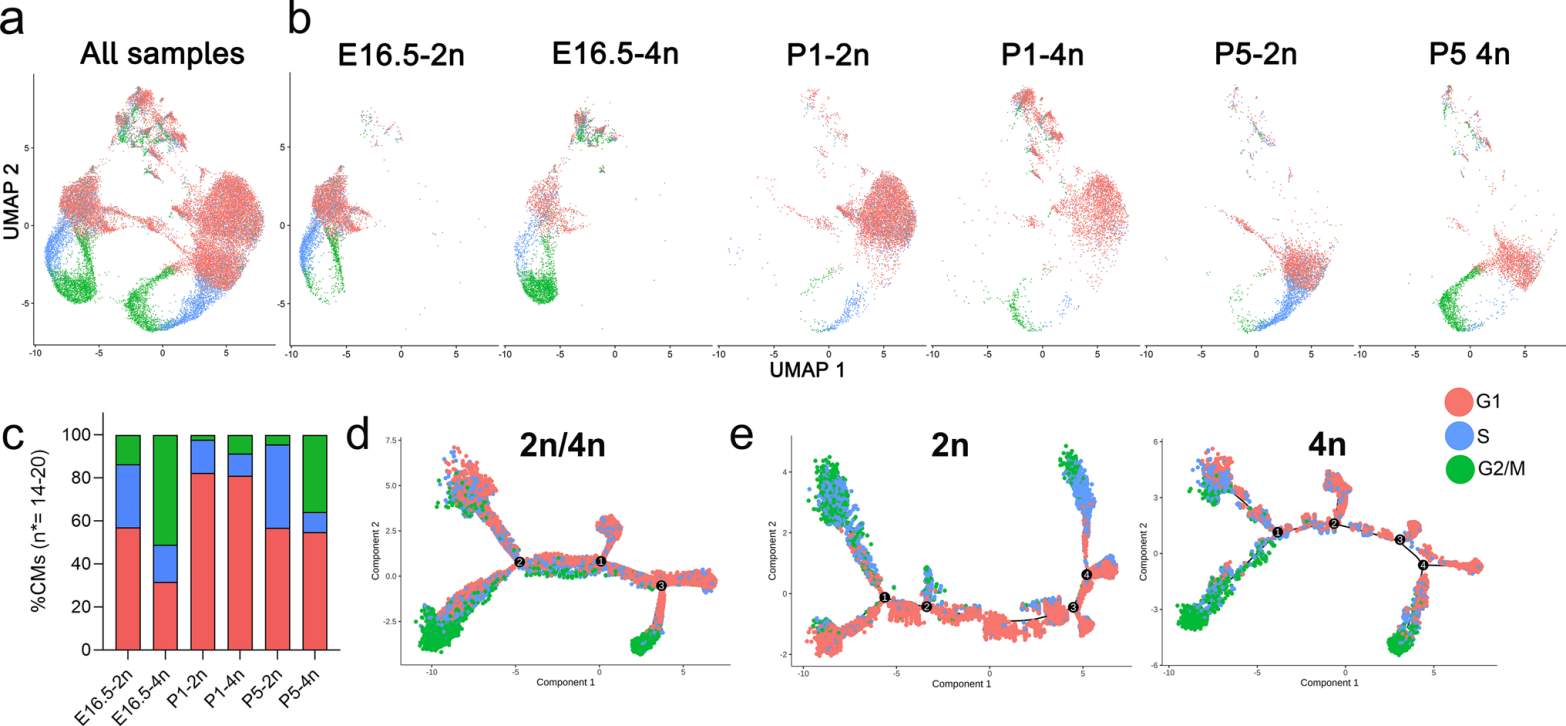
**Distribution of CMs within cell cycle phases.** **a**, **b**, UMAP plots of CMs with visualization of the cell cycle status across developmental stages and ploidy, **c**, Quantification of cells representing each cell cycle phase (n* = number of animals). **d, e,** Trajectory analysis of CMs (**d** 2n and 4n pooled; **e** divided into 2n and 4n)) across developmental stages with cell cycle phase identity marked

To further explore the cell cycle status of the identified CM clusters (Fig. 3a), we used gene signatures that have previously been shown to denote G1, S, and G2/M phases [67]. Thus, whereas ploidy was assigned by FACS prior to scRNA-seq, separation according to cell cycle identity was performed bioinformatically. Yet, as the G2 and M phases cannot be readily distinguished based on gene expression they are considered united as “G2/M”. As such, we found that the two “Cell division” clusters (Fig. 2a, b) were composed of CMs active in either the S-phase or G2/M-phases (Fig. 2a, b) whereas the CMs composing the “Fatty acid metabolic process”-cluster (Fig. 2a, b) were mainly ascribed to the G1-phase (Fig. 3a, b). The percentage of predicted G1-phase CMs was fairly constant between ploidies at P1 (2*n* = 82.3%; 4*n* = 81.1%) and P5 (2*n* = 56.9%; 4*n* = 54.9%) but differed at the embryonic stage E16.5 (2*n* = 57%; 4*n* = 31.8%) (Fig. 3c). These percentages also imply that a relatively higher percentage of cells are in active cell cycle (not G1) at E16.5 and at P5 as compared to P1 in agreement with the GO term analysis (Fig. 2b). In consensus with biology, the majority of G2/M CMs were mainly found in the tetraploid portion of CMs, particularly the E16.5-4n CMs (2*n* = 13.6%; 4*n* = 51%) and to some extent the P5-4n CMs (2*n* = 4.4%; 4*n* = 35.9%) (Fig. 3c). In contrast, G2/M CMs were scarce at P1 (2*n* = 2.2%; 4*n* = 8.7%) (Fig. 3c). Inversely, S-phase CMs were more pronounced in diploid CMs (E16.5: 2*n* = 29.3%; 4*n* = 17.2%, P1: 2*n* = 15.4%; 4*n* = 10.2%, P5: 2*n* = 38.7%; 4*n* = 9.2%) (Fig. 3c). This agrees with cell cycle active diploid CMs mainly being in S-phase, while tetraploid CMs have progressed to the G2/M phases. In pseudotime, S-phase CMs manifested in the start of the trajectory, but some did align broadly throughout pseudotime as did also G1 phase CMs (Figs. 2d and 3d, e; Supplementary Fig. 2a). In contrast, the large cluster of 4n E16.5-G2/M CMs confined to the two earliest branches in pseudotime, whereas P5-G2/M CMs mainly located to the downward bifurcation in branchpoint 3, which represented more mature CMs characterized by "Fatty acid metabolism” and “Muscle contraction” (Fig. 2e). These data thus supported that E16.5-4n-G2/M CMs likely represented dividing CMs whereas the more mature P5-4n-G2/M CMs were in the process of polyploidization/binucleation, which agrees with CM proliferation being absent after P3 [61] and our combined FACS and microscopic analyses described above (Fig. 1b, c and Supplementary Fig. 1e, f).

**Fig. 4:**
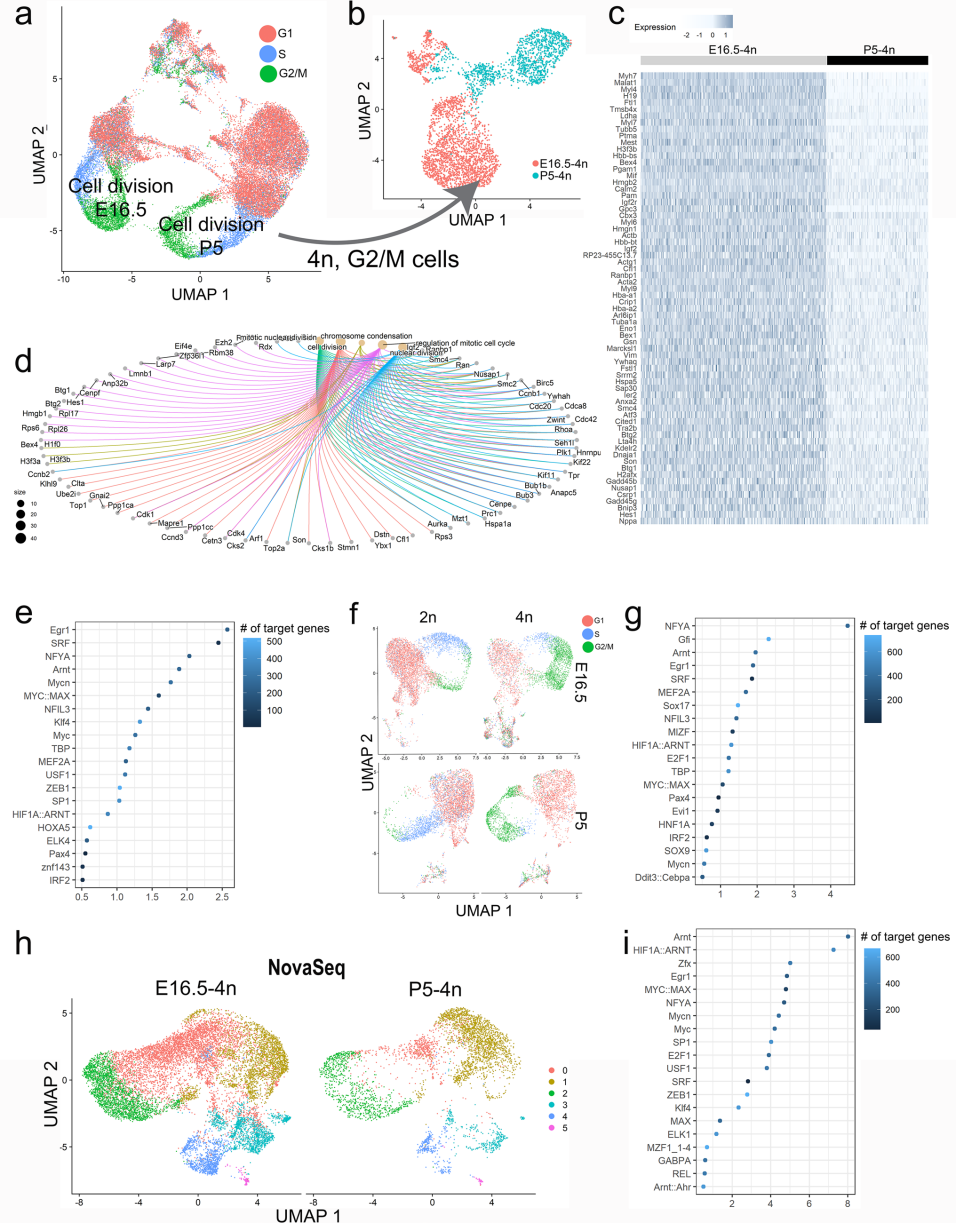
**Enriched set of transcription factors (TFs) specific for CM G2/M progression.** **a** UMAP plot of 2n- and 4n CMs at E16.5, P1, and P5 with cell cycle phase identity visualized. **b** UMAP plot of extracted G2/M, 4n CMs from E16.5 and P5 developmental stage from the two Cell division clusters as indicated in (**a**). **c** Heatmap of expression of top genes with increased expression in E16.5 CMs compared to P5 CMs in (**b**). **d** cnetplot of selected cell-cycle related GO terms enriched among the genes in (**c**). **e** Enriched TFs (oPOSSUM) for genes with increased expression in E16.5 CMs compared to P5 CMs in (**b**). **f** UMAP plot of 2n- and 4n CMs at E16.5 and P5 separately analyzed with cell cycle phase identity visualized. **g** Enriched TFs (oPOSSUM) for genes with increased expression in E16.5 CMs compared to P5 CMs in (**f)**. **h** UMAP plot of re-sequenced data with NovaSeq. Clustering is indicated and CMs separated according to developmental stage (E16.5 and P4, both 4n). **i** Enriched TFs (oPOSSUM) for genes with increased expression in E16.5 CMs compared to P5 CMs in (**h**)

Then to identify which TFs that regulate the G2/M phases in immature CMs (E16.5) as compared to those that halt before karyokinesis/cytokinesis (P5), we extracted CMs composing the two “Cell division” clusters (Figs. 2a and 4a) and focused the analysis on tetraploid CMs ascribed to the G2/M-phases extracting a subset of the data for further analysis (Fig. 4b). Then, we obtained a list of marker genes with higher expression in E16.5-4n-G2/M-CMs as compared to P5-4n-G2/M-CMs (Fig. 2c) and performed subsequent GO term analysis. Several terms related to the cell cycle: “Mitotic nuclear division”, “Cell division”, “Chromosome condensation”, “Regulation of mitotic cell cycle”, and “Nuclear division” and associated genes were unraveled by a gene concept network map (Fig. 4d). Using the oPOSSUM platform, enriched TF binding sites were determined for the gene expression enriched in E16.5-4n-G2/M CMs (Fig. 4b, c), and allowed us to predict a list of TFs potentially regulating G2/M progression in dividing CMs (Fig. 4e). To confirm this list of TFs and avoid the initial selection of the “Cell division” clusters, which could bias the approach, we combined E16.5- and P5-CMs into a new dataset and reanalyzed with specific identification of E16.5-4n-G2/M CMs and P5-4n-G2/M CMs (Fig. 4f). This repeated analysis resulted in a second list of TFs (Fig. 4g) very similar to that obtained for the cluster-based approach (Fig. 4e). Moreover, to exclude that the identified set of TFs (Fig. 4e, g) reflected data of low sequence saturation, we performed NovaSeq (mean reads per CM were amplified 4.5- and 8.3-fold for E16.5-4n and P5-4n CMs, respectively) with an increase from 13,578 to 16,118 in the total number of genes detected (Supplementary Fig. 3a–c). Together, these analyses supported that the identified TFs likely play a role in regulating G2/M of dividing CMs. Based on the Fisher score (Fig. 2e, g, i), we therefore selected *NFYA, SRF, MYC::MAX, USF1, MYCN, EGR1, ARNT, MYC, E2F1,* and *ZEB1* for further analysis. We also included *SP1* for further analysis as this TF has been associated with CM cell cycling previously [21]. Since, MYC::MAX represents a complex of the two individual TFs, MYC and MAX, we therefore proceeded with 11 TFs in total for further analysis.

### ScRNA-seq identified TFs that affect CM cell cycling

Since, expression of TFs often are relatively low and given their nature as being upstream regulators of their target genes, detection of TF expression in scRNA-seq data are often complicated. Despite these challenges, our CM scRNA-seq data did reveal several CMs expressing the identified TFs. Yet, the percentages, of CMs expressing the TFs were still quite small, and their levels were decreasing with developmental stage (Supplementary Figs. 4 and 5).

**Fig. 5:**
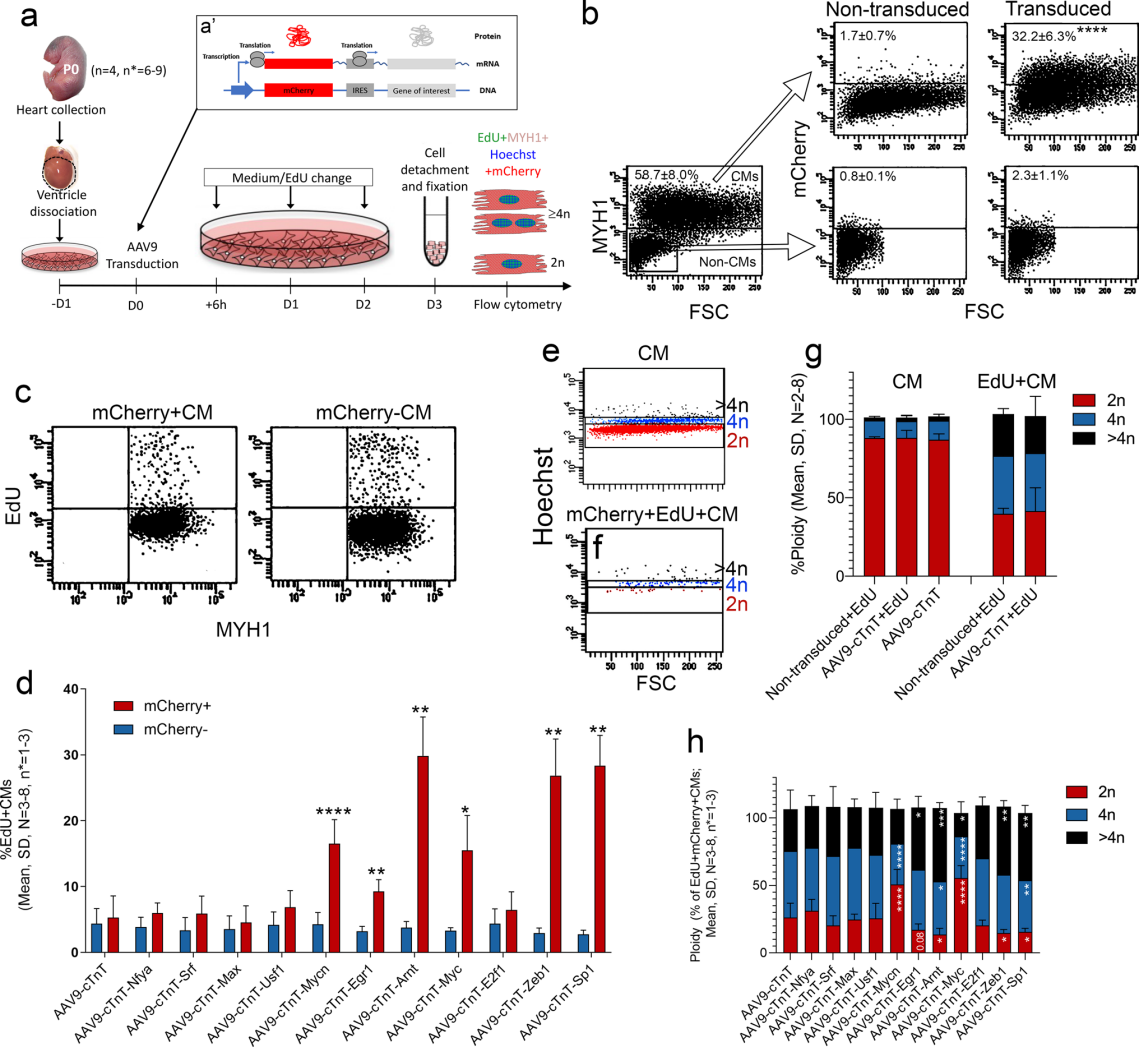
**ScRNA-seq identified transcription factors (TFs) regulate CM cell cycle activity. a** Schematic of the study design and workflow. Neonatal heart cells were isolated at P0 and cultured for 24 h, before transductions with AAV9-cTnT-TF. Insert depicts as modified from Addgene. EdU incorporation into mCherry^+^ and mCherry^−^ CMs together with CM- (MYH1^+^) and ploidy- (Hoechst) identity were then assessed by flow cytometry. **b** Flow cytometric dot plots of non-transduced and transduced heart cells cultured for 3 days after viral transduction showing CM specificity for AAV9-cTnT-TF expression (two-way ANOVA with Sidak’s post test, ****P ≤ 0.0001). **c-d** EdU incorporation in mCherry^+^ (TF transduced) and mCherry^−^ (non-transduced of the same culture) CMs quantified by flow cytometry. The percentage of EdU^+^mCherry^+^ CMs was compared to that of empty vector transduced CMs (AAV9-cTnT) (ANOVA and Fisher’s LSD post test, *N* = 3–8, *P ≤ 0.05, **P ≤ 0.01, ***P < 0.001 and ****P ≤ 0.0001) or to the % EdU^+^mCherry^−^ CMs within the culture (Data not shown). **e–h** Flow cytometric assessment of the percentage of diploid- (2n), tetraploid- (4n) and polyploid- (> 4n) transduced and non-transduced CMs as indicated (ANOVA and Fisher’s LSD post test, *P ≤ 0.05, **P ≤ 0.01, ***P < 0.001 and ****P ≤ 0.0001). Gates were defined using all CMs in (**e**)

Thus, to evaluate the biological effect of the 11 TFs on CM cell cycle activity we overexpressed them in P0 cardiac cells (CM^P0^). To mimic the in vivo heart environment, we initially used mixed cardiac cell cultures rather than a pure CM population. Yet, to enable CM specific TF overexpression and identification of transduced CMs, we generated CM specific (cTnT promoter) adeno associated viruses of serotype 9 (AAV9) with a mCherry reporter and an internal ribosome entry site (IRES) for each of the 11 selected TFs (denoted AAV9-cTnT-TF) (Fig. 5a). Accumulated cell cycle activity in CM^P0^ was measured 72 h after AAV9-cTnT-TF transductions using flow cytometry for EdU/MYH1/mCherry/Hoechst (Fig. 5a). Virus functionality for each of our AAV9-cTnT-TFs was validated by mCherry and TF mRNA expression (Supplementary Fig. 6a, c), whereas flow cytometry for mCherry^+^ CMs confirmed transduction efficiencies (Supplementary Fig. 6b). Both mCherry mRNA and reporter fluorescence showed that the efficiency of TF expression decreased with the increasing size of the TF (Supplementary Fig. 6a, c, d). To counterbalance we increased the number of CMs analyzed by flow cytometry for large-sized TFs. Moreover, to ensure a high biological diversity and account for a 12 h difference in birth delivery, each independent experiment embraced CM^P0^ pooled from one litter each constituting 6–9 mice. At the time of analysis, 72 h after transduction, CM^P0^ cultures consisted of 58.7 ± 8.0% CMs (mean, SD, *n* = 8), and AAV9-cTnT-TF expression was highly specific for CMs (Fig. 5b). The percentage of EdU^+^ CM^P0^ transduced with an empty AAV9-cTnT-mCherry vector did not differ between mCherry^+^ and mCherry^−^ CMs (5.3 ± 3.3% versus 4.4 ± 2.3%; mean, SD, *n* = 8; *p* = 0.9992; Adjusted p-value, Sidak test) (Fig. 5c, d), suggesting that transduction did not alter S-phase progression in itself. This also verified that cell cycle activity indeed is relatively low per se in CM^P0^ cultures. When compared to empty control transduced mCherry^+^ CM^P0^ (5.3 ± 3.3%), *Mycn* (16.5 ± 3.6%), *Egr1* (9.3 ± 1.8%), *Arnt* (29.8 ± 5.9%), *Myc* (15.5 ± 5.3%), *Zeb1* (26.8 ± 5.6%), and *Sp1* (28.4 ± 4.6%) overexpression resulted in significant increases in the percentage of EdU^+^mCherry^+^ CMs (Fig. 5d). This was consistent when comparing EdU^+^mCherry^+^ CMs with EdU^+^mCherry^−^ CMs within each culture (Fig. 5d). Thus, overexpression of TFs corresponded to 3.1- (*Mycn*), 1.8- (*Egr1*), 5.6- (*Arnt*), 2.9- (*Myc*), 5.1- (*Zeb1*), and 5.4-fold (*Sp1*) inductions in the percentage of EdU^+^ CMs. The remaining TFs did not significantly alter the percentage of EdU^+^mCherry^+^ CMs neither as compared to empty control nor as compared to EdU^+^ mCherry^−^ CMs (Fig. 5d). Thus, the six TFs *Mycn*, *Egr1*, *Arnt*, *Myc*, *Zeb1*, and *Sp1* were able on their own to increase cell cycle activity of CM^P0^. However, it is generally acknowledged that EdU incorporation only reflects whether a given CM has progressed through the S-phase and therefore does not reflect if the CM completed cell division [37, 55]. We therefore assessed ploidy in the transduced CMs and defined borders for a ploidy of either 2n, 4n, and > 4n (Fig. 5e), and used those gates for analyzing EdU^+^mCherry^+^ CMs (Fig. 5f). Neither EdU incorporation nor AAV9-cTnT transduction affects CM ploidy in themselves (Fig. 5g) ensuring the validity of the analysis. Using this setup, we found a significant change in ploidy for EdU^+^mCherry^+^ CMs overexpressing *Mycn*, *Egr1*, *Arnt*, *Myc*, *Zeb1*, and *Sp1*, whereas no change in ploidy was observed for the TFs also not altering the percentage of EdU^+^mCherry^+^ CMs (Fig. 5h). *Myc* and *Mycn* overexpression clearly resulted in EdU^+^mCherry^+^ CMs being diploid suggesting completed cytokinesis and thus proliferation. In contrast, *Egr1*, *Arnt*, *Zeb1*, and *Sp1* favored polyploidy at the expense of diploid CMs (Fig. 5h) indicating karyokinesis/cytokinesis failure.

We next subjected the target genes (according to oPOSSUM 3.0) of our six identified TFs to GO term analysis (*p* < 0.05, Benjamini corrections) and found that 4- (MYC), 5- (MYCN), 3- (EGR1), 3- (ARNT), 13- (ZEB1), and 4- (SP1) out of 13 GO terms related to “Cell cycling/division” (3), “Mitotic division/cytokinesis” (4), “Telomers” (3), and “Regulation of cell cycling”/”Positive regulation of cell proliferation” (3) (Supplementary Fig. 7). When adjusting for gene overlap between the different cell cycle related GO terms, 53- (MYC), 60- (MYCN), 41- (EGR1), 45- (ARNT), 118- (ZEB1), and 72- (SP1) genes related to these cell cycle related GOs were affected by the given TF (Supplementary Fig. 7).

Overall, these data underscore the design and quality of our CM scRNA-seq data, and enabled us to identify at least six TFs regulating cell cycle activity of CMs, where two (*Myc, Mycn*) and four (*Egr1*, *Arnt*, *Zeb1*, and *Sp1) TFs* resulted in cytokinesis or a lack hereof, respectively. Since the *Zinc Finger E-Box Binding Homeobox 1* gene (*Zeb1*) was superior to the other TFs in regulating the number (118) of target genes associated to cell cycle activity in CMs, we hypothesized that ZEB1 could be a novel key player in CM proliferation.

### ZEB1 identified as a novel regulator of G2/M progression and CM proliferation before birth

**Fig. 6:**
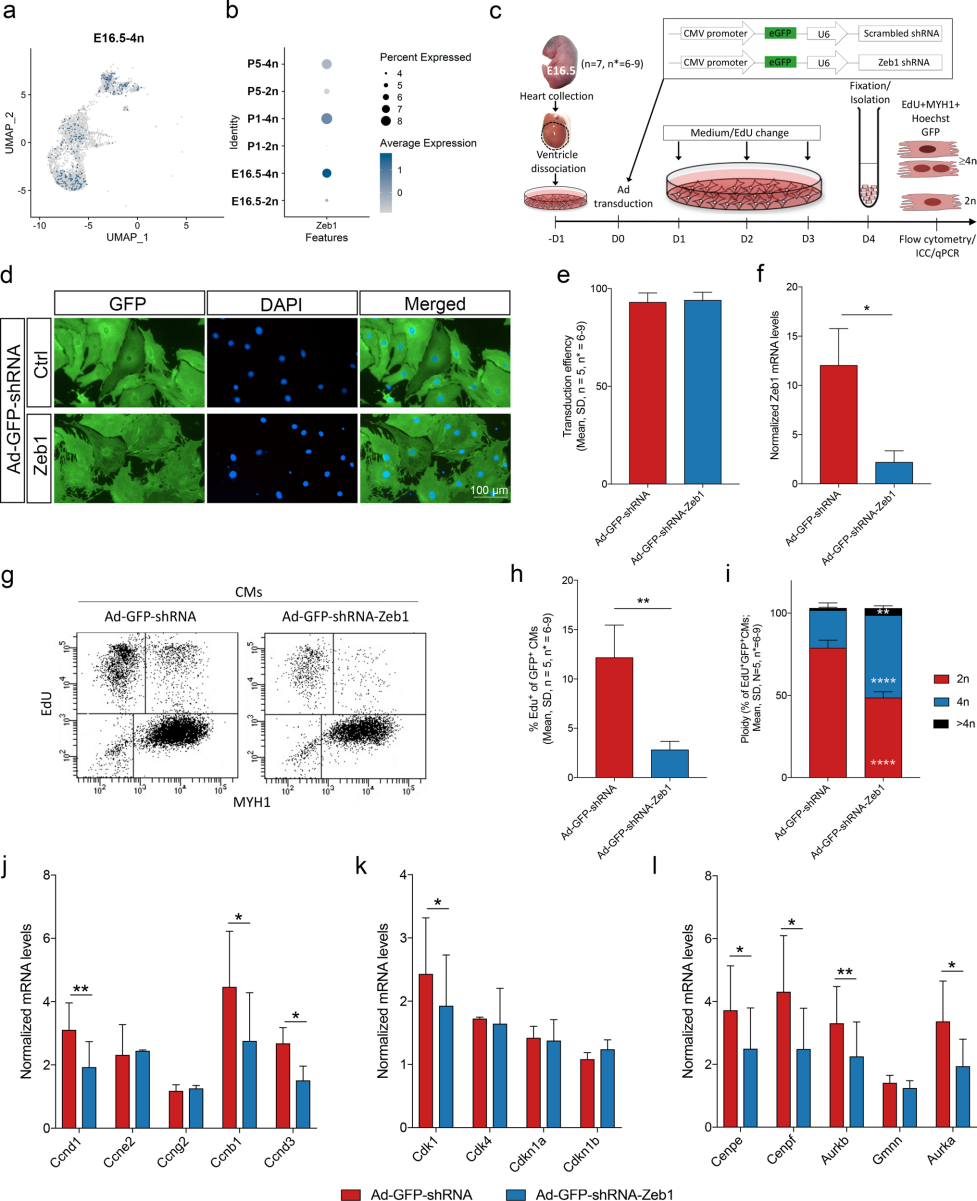
**ZEB1 knockdown reduce CM proliferation before birth.** **a** UMAP plot of E16.5-4n CMs extracted from UMAP plot of all six conditions (as in Fig. 2a) with blue color indicating CMs expressing *Zeb1* mRNA. **b** Dot plot of *Zeb1* expression for all six conditions with size indicating percentage of CMs expressing *Zeb1* and color indicating the average expressing level in *Zeb1*-expressing CMs. **c** Schematic of the study design and workflow. Embryonic hearts were isolated at E16.5 and cells were cultured for 24 h before adenoviral transduction with either Ad-GFP-shRNA (control; scrambled shRNA) or Ad-GFP-shRNA-Zeb1. The insert depicts the viral construct containing an eGFP reporter. Cells were fixed 96 h after transduction and EdU pulsing, and analyzed by flow cytometry and immunocytochemistry (ICC). **d** ICC of E16.5 primary cardiac cultures transduced with either Ad-GFP-shRNA or Ad-GFP-shRNA-Zeb1 after 96 h (GFP^+^, green; DAPI (cell nuclei), blue). **e** GFP expression quantified by flow cytometry of Ad-GFP-shRNA and Ad-GFP-shRNA-Zeb1 transduced cells 96 h after transduction (Paired *t*-test, *n* = 5, NS). **f** Normalized mRNA level of ZEB1 in Ad-GFP-shRNA and Ad-GFP-shRNA-Zeb1 transduced cells (normalized against *B2m* and *Gapdh*; Paired *t*-test, *n* = 3, *P ≤ 0.05). **g** Flow cytometry dot plot of EdU incorporation in MYH^+^ cells transduced with Ad-GFP-shRNA or Ad-GFP-shRNA-Zeb1. **h** EdU positive CMs transduced with Ad-GFP-shRNA or Ad-GFP-shRNA-Zeb1 quantified by flow cytometry (Paired *t*-test, *n* = 5, **P ≤ 0.01). **i** CM ploidy of Ad-GFP-shRNA and Ad-GFP-shRNA-Zeb1 transduced, EdU positive cells quantified by flow cytometry based on Hoechst intensity (Paired *t*-test, *n* = 5, **P ≤ 0.01; ****P ≤ 0.0001). **j** Normalized mRNA levels of *Ccnd1*, *Ccne2*, *Ccng2*, *Ccnb1*, and *Ccnd3* (normalized against *B2m* and *Gapdh*; Paired *t*-test, *n* = 3, *P ≤ 0.05; **P ≤ 0.01). **k** Normalized mRNA levels of *Cdk1*, *Cdk4*, *Cdkn1a*, and *Cdkn1b* (normalized against *B2m* and *Gapdh*; Paired *t*-test, *n* = 3, *P ≤ 0.05). **l** Normalized mRNA levels of *Cenpe*, *Cenpf*, *Aurkb*, *Gmnn*, and *Aurka* (normalized against *B2m* and *Gapdh*; Paired *t*-test, *n* = 3, *P ≤ 0.05; **P ≤ 0.01)

By reassessing the literature, the role of ZEB1 appears poorly described in the heart and CMs. In a study by Riechert and coworkers, it was found that Zeb1 is involved in CM hypertrophy [54] and Cencioni et al. showed that the Zeb1-Hdac2-eNOS circuity identifies early cardiovascular precursors [9]. However, the role of ZEB1 in CM proliferation and polyploidization remains elusive. Interestingly, Zeb1 null mice do not survive postnatally due to respiratory failure and T-cell deficiency [64]. Reinvestigating our scRNA-seq data we found that ZEB1 was present in only a small fraction of CMs (Fig. 6a, Supplementary Fig. 4) preferably in the G2/M phases (Fig. 6b), but the level declined with developmental stage (Supplementary Fig. 5). To investigate the implications of Zeb1 in CM proliferation before birth, ZEB1 knockdown was performed using adenoviral transduction for administration of validated Zeb1 shRNA in E16.5 primary heart cultures (Fig. 6c). A high transduction efficiency was confirmed in CMs (Fig. 6d) with no significant difference between control Ad-GFP-shRNA- (93.1 ± 4.7%; mean, SD, *n* = 5) and Ad-GFP-shRNA-Zeb1 treatments (94.2 ± 4.0%; mean, SD, *n* = 5) (Fig. 6e), and overall ZEB1 was reduced by 82.3 ± 5.9% (mean, SD, *n* = 3)(Fig. 6f). Using this setup, we found that cell cycle activity in CMs as reflected by EdU incorporation was decreased significantly in Ad-GFP-shRNA-Zeb1 treated CMs from 12.2 ± 3.3% to 2.8 ± 0.8% (mean, SD, *n* = 5)(Fig. 6g,h). Importantly, the diploid status of EdU^+^ /CMs decreased by Zeb1 knockdown (Fig. 6i) suggesting that not only S-phase progression was inhibited, but also cytokinesis was reduced by Zeb1 knockdown. This was supported by a significant downregulation, in Ad-GFP-shRNA-Zeb1 treated cells, of *Ccnd1* (Cyclin D1), *Ccnb1* (Cyclin B1) and *Ccnd3* (Cyclin D3), while the levels of *Ccne2* (Cyclin E2) and *Ccng2* (Cyclin G2) associated with endoreplication [48, 82] were unchanged (Fig. 6j). Although, the level of the major cyclin dependent kinase, *Cdk1* (Cyclin-dependent kinase 1), was slightly reduced when Zeb1 was knocked down (Fig. 6k), no difference was observed for *Cdk4* (Cyclin-dependent kinase 4) and the cell cycle inhibitors *Cdkn1a* (Cyclin-dependent kinase inhibitor 1a, *p21*) and *Cdkn1b* (Cyclin-dependent kinase inhibitor 1b, *p27*) (Fig. 6k). With a focus on factors known from the G2/M phase, where Zeb1 predominantly was observed (Fig. 6b), we found a significant increase in the expression of *Cenpe* (Centromere protein E), *Cenpf* (Centromere protein F), *Aurkb* (Aurora kinase B), and *Aurka* (Aurora kinase A), but no change in the level of *Gmnn* (Geminin) (Fig. 6l). These data confirm the scRNA-seq data that ZEB1 mediates the cell cycle program and seems required for CM proliferation before birth.

### ZEB1 overexpression leads to CM endoreplication and high ploidy after birth

**Fig. 7:**
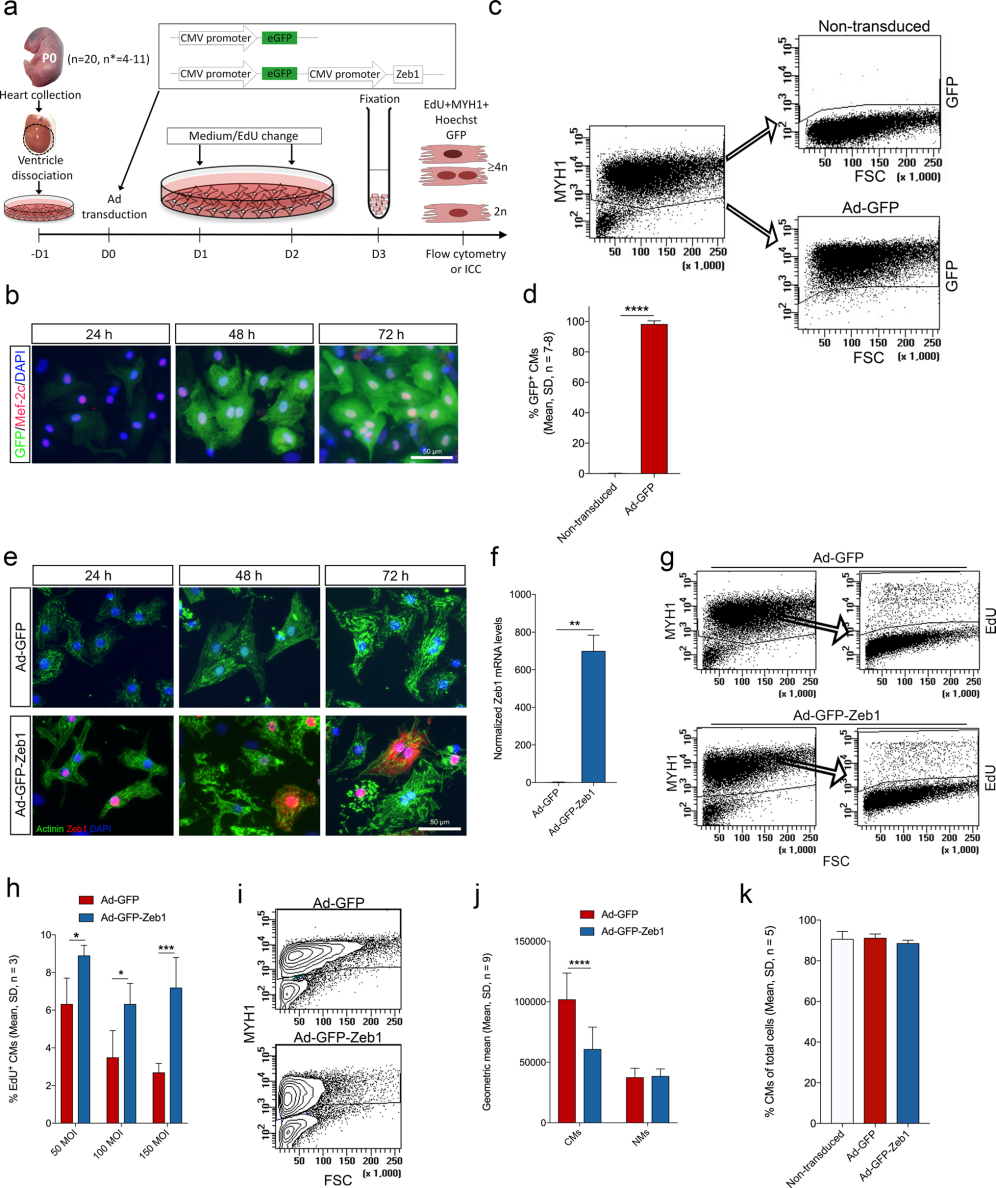
**ZEB1 increases ploidy and decreases cell size in vitro.** **a** Schematic of the study design and workflow. Neonatal hearts were isolated at P0 and cultured for 24 h before transduction with adenovirus (Ad). The insert depicts the viral construct, note the eGFP reporter in both Ads. Cells were fixed after 72 h transduction and EdU subjection and analyzed by flow cytometry or immunocytochemistry (ICC). **b** ICC of Ad-GFP transduced primary CMs after 24, 48, and 72 h of transduction (GFP^+^, green; Mef-2c^+^ (CM nuclei), red; DAPI (cell nuclei), blue). **c** Flow cytometric dot plots of GFP^+^ and GFP^−^ MYH1^+^ CMs in non-transduced and Ad-GFP transduced CMs. **d** GFP expression quantified by flow cytometry after 72 h of culturing (non-transduced) or Ad-GFP transduction (Paired *t*-test, *n* = 8, ****P ≤ 0.0001). **e** ICC of Ad-GFP (top panel) and Ad-GFP-Zeb1 (lower panel) transduced CMs after 24, 48, and 72 h transduction (actinin^+^ (CMs), green; ZEB1^+^, red; DAPI (cell nuclei), blue). **f** *Zeb1* expression in CMs after 72 h Ad-GFP or Ad-GFP-Zeb1 transduction as quantified by qRT-PCR (normalized against *B2m* and *Rpl_13A*; Unpaired *t*-test, *n* = 6–7, **P ≤ 0.01). **g** Flow cytometric dot plots of EdU incorporation in MYH1^+^ CMs after 72 h Ad-GFP transduction (top panel) and Ad-GFP-Zeb1 transduction (lower panel) (forward side scatter (FSC)). **h** Incorporation of EdU was observed at both 50, 100, and 150 MOI after 72 h Ad-GFP and Ad-GFP-Zeb1 transduction (Unpaired t-test, *n* = 3, *P ≤ 0.05, ***P ≤ 0.001). **i** Flow cytometric contour plots depicting decreased MYH1^+^ CM size after 72 h Ad-GFP-Zeb1 transduction. **j** Quantification of the geometric mean of CMs and non-myocytes (NMs) 72 h after Ad-GFP or Ad-GFP-Zeb1 transduction (Two-way ANOVA, *n* = 9, ****P ≤ 0.0001). **k** Percentages of CMs of the total cell number after 72 h in culture (non-transduced), or after 72 h Ad-GFP or Ad-GFP-Zeb1 transduction (One-way ANOVA followed by Tukey’s multiple comparisons test, *n *= 5)

**Fig. 8:**
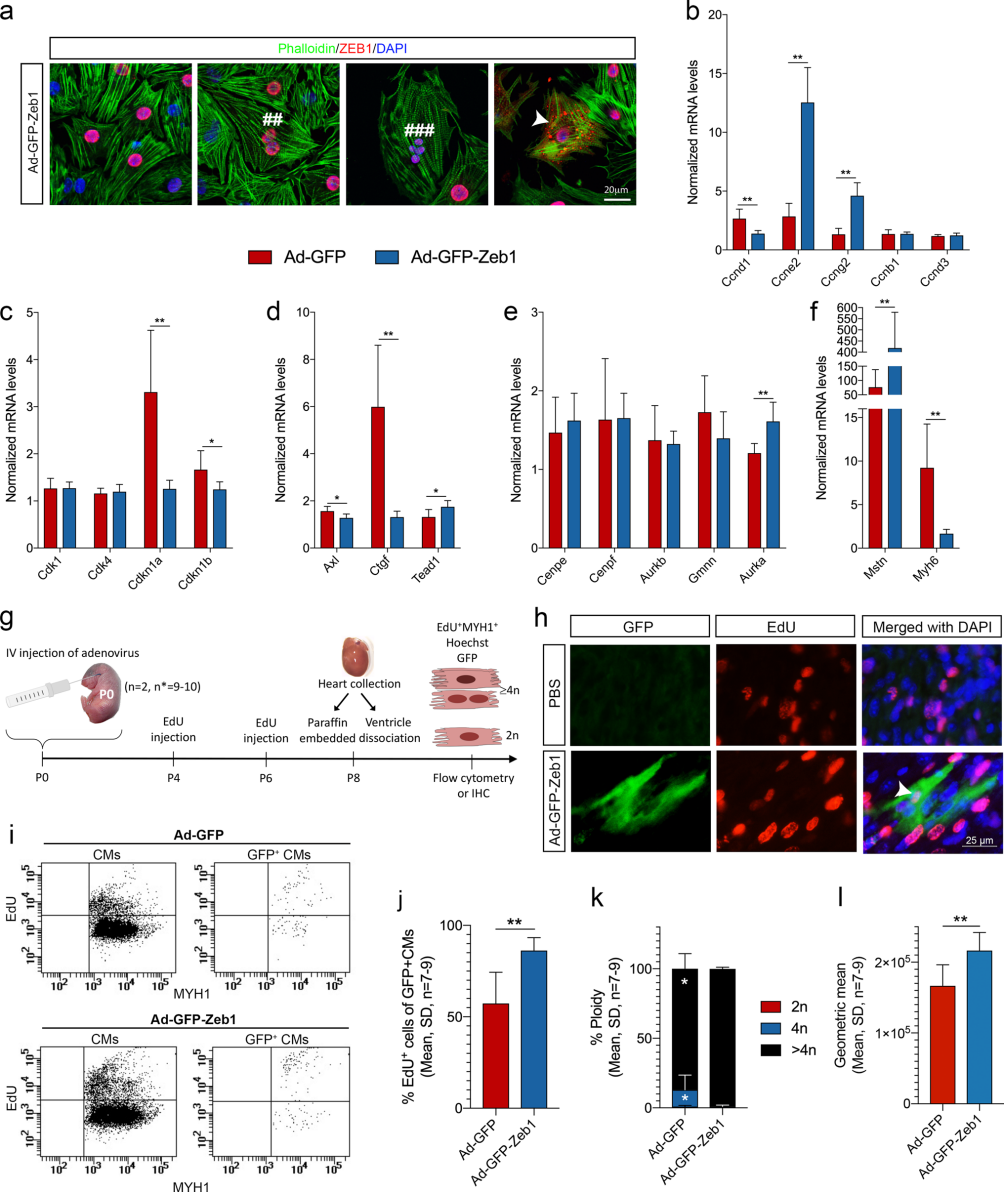
**ZEB1 increase ploidy and cell size in vivo.** **a** Confocal images of Ad-GFP-Zeb1 transduced CMs after 72 h transduction (phalloidin (F-actin) green; ZEB1^+^, red; DAPI (cell nuclei), blue). Binucleated CM (##), tetranucleated CM (###), cytoplasm localized ZEB1 (arrowheads). **b** Normalized mRNA levels of *Ccnd1*, *Ccne2*, *Ccng2*, *Ccnb1*, and *Ccnd3* (normalized against *B2m* and *Rpl_13A*; Unpaired *t*-test or Mann–Whitney based on normality, *n* = 6–7, **P ≤ 0.01). **c** Normalized mRNA levels of *Cdk1*, *Cdk4*, *Cdkn1a*, and *Cdkn1b* (normalized against *B2m* and *Rpl_13A*; Unpaired t-test or Mann–Whitney based on normality, *n* = 6–7, **P ≤ 0.01). **d** Normalized mRNA levels of *Axl*, *Ctgf*, and *Tead1* (normalized against *B2m* and *Rpl_13A*; Unpaired *t*-test or Mann–Whitney based on normality, *n* = 6–7, *P ≤ 0.05, **P ≤ 0.01). **e** Normalized mRNA levels of *Cenpe*, *Cenpf*, *Aurkb*, *Gmnn*, and *Aurka* (normalized against *B2m* and *Rpl_13A*; Unpaired t-test or Mann–Whitney based on normality, *n* = 6–7, *P ≤ 0.05). **f** Normalized mRNA levels of *Mstn*, and *Myh6* (normalized against *B2m* and *Rpl_13A*; Unpaired *t*-test or Mann–Whitney based on normality, *n* = 6–7, **P ≤ 0.01). **g** Schematic of the study design and workflow. Neonatal pups (P0) were injected with Ad-GFP or Ad-GFP-Zeb1 through the superficial temporal vein, followed by two subcutaneous injections of EdU at P4 and P6. Hearts were collected at P8 for immunohistochemistry (IHC) or ventricular dissociation for flow cytometry. **h** IHC of P8 heart ventricle tissue from PBS (top panel, PBS injected pups were included in the study with the purpose to make this figure to show the GFP-signal in adenovirus-injected pups compared to PBS-injected pups) or Ad-GFP-Zeb1 (lower panel) P0 injected pups (GFP^+^, green; EdU^+^, red; DAPI (cell nuclei), blue). **i** Flow cytometric dot plots depicting the EdU incorporation in all MYH1^+^ CMs (left panel) as well as in MYH1^+^GFP^+^ CMs (right panel) after 72 h Ad-GFP (top panel) or Ad-GFP-Zeb1 (lower panel) transduction. **j** Percentages EdU^+^GFP^+^ CMs of GFP^+^ CMs at P8 after Ad-GFP injection at P0 compared to Ad-GFP-Zeb1 injection (Paired *t*-test, *n* = 7–9, **P ≤ 0.01). **k** Percentage-wise distribution of ploidy at P8 after Ad-GFP or Ad-GFP-Zeb1 injection at P0 (Paired *t*-test, *n* = 7–9, *P ≤ 0.05). **l** Quantification of the geometric mean of primary CMs at P8 after Ad-GFP or Ad-GFP-Zeb1 transduction (Unpaired *t*-test, *n* = 7–9, **P ≤ 0.01)

With Zeb1 as a key player in CM proliferation before birth, we then investigated if reintroducing ZEB1 at high efficiency in postnatal CMs could override terminal CM differentiation and lead to CM proliferation after birth. To overcome the observed challenge of AAV9-TF transduction effectivity of the large-sized *Zeb1* (Supplementary Fig. 6d), we generated adenovirus (Ad) with an eGFP reporter to be expressed separately from ZEB1 and used that for inducing ZEB1 expression in CM^P0^ (Fig. 7a). As visualized by immunocytochemistry (Fig. 7b) and quantified by flow cytometry (Fig. 7c, d), Ad-GFP CM transduction efficiency was 98.22 ± 2.2% at 72 h with a parallel and specific expression of ZEB1 (Fig. 7e, f, Supplementary Fig. 8a, b). In agreement, with its nature as a TF, the overexpressed ZEB1 protein mainly localized to the nucleus in CMs (Fig. 7e, Supplementary Fig. 8a, b). We did, however, not observe ZEB1 associated to the tubulin spindle apparatus (Supplementary Fig. 8b) as noted in cancer cell lines [16], but we did notice a few scattered CMs with cytoplasmic ZEB1 (Fig. 7e and Supplementary Fig. 8b). EdU pulse chase labelling with empty virus Ad-GFP and Ad-GFP-Zeb1 supported the above experiments showing that the percentage of EdU^+^ CMs increased significantly with ZEB1 overexpression (Fig. 7h). Moreover, a massive reduction in CM size was apparent after 72 h of ZEB1 overexpression, and did not occur in non-CMs (Fig. 7i, j). No difference was observed in the percentage of CMs between non-transduced, Ad-GFP, and Ad-GFP-Zeb1 cultures at 72 h after transduction (Fig. 7k), suggesting that CM death or apoptosis was minimal at this timepoint and was not induced by virus treatment. ZEB1 overexpression revealed both single-, bi-, and multinucleated CMs at 72 h after transduction (Fig. 8a and Supplementary Fig. 8b), supporting the observed ability to induce CM S-phase progression (Figs. 5d and 7h) leading to polyploidy and not division (Fig. 5h). In contrast to our Ad-GFP-shRNA-Zeb1 studies (Fig. 6j), we observed a downregulation of *Ccnd1* with a concomitant upregulation of *Ccne2* and *Ccng2*, while the levels of *Ccnb1* and *Ccnd3* were unchanged (Fig. 8b). No change was observed for *Cdk1* and *Cdk4*, whereas *Cdkn1a* was dramatically reduced. Likewise, *Cdkn1b* was reduced, but to a lesser extent (Fig. 8c). We also evaluated the Hippo pathway known from cardiac regeneration through CM proliferation, and found its downstream targets, *Axl* (Axl receptor tyrosine kinase) and *Ctgf* (connective tissue growth factor) to be reduced in Ad-GFP-Zeb1 transduced CMs, with a slight increase in *Tead1* (TEA domain family member 1) (Fig. 8d). Also, in contrast to Ad-GFP-shRNA-Zeb1 treatments (Fig. 6), there was no change in expression of the G2/M phase factors *Cenpe*, *Cenpf*, *Aurkb*, and *Gmnn* besides a minor increase in *Aurka* (Aurora kinase A) levels (Fig. 8e). Additionally, we found that the muscle size inhibitor *Mstn* (Myostatin) were substantially increased in ZEB1 overexpressing CMs, while the *Myh6 * expression was substantially reduced (Fig. 8f), which may explain why the smaller size of CMs was observed early after ZEB1 overexpression (Fig. 7i,j). Finally, we injected Ad-GFP and Ad-GFP-Zeb1 into the superficial temporal vein of P0 mice to evaluate ZEB1 overexpression directly in the heart, and analyzed by EdU incorporation, its effect on CM cell cycling from P0 to P8 (Fig. 8g). Scattered transduced GFP^+^ CMs were found in all hearts of Ad injected pups (Fig. 8h), and as estimated by flow cytometry 1.5–2.5% of CMs were GFP^+^ at P8 (Fig. 8i). EdU incorporation was visualized in Ad-GFP-Zeb1 transduced CMs (Fig. 8h), and upon quantification by flow cytometry a significant higher percentage of EdU^+^ CMs were found in Ad-GFP-Zeb1 transduced CMs as compared to Ad-GFP transduced CMs (Fig. 8j) confirming the in vitro results with increased cell cycle activity as a result of Zeb1. Moreover, 99 ± 1% of the EdU^+^/Ad-GFP-Zeb1^+^ CMs exhibited a ploidy of > 4n, while the remaining 1% were 4n, which was different from EdU^+^/Ad-GFP^+^ CMs showing lower ploidy (Fig. 8k). The “high ploidy” Ad-GFP-Zeb1 transduced CMs were 30% larger than their Ad-GFP^+^ CM counterparts at P8 (Fig. 8l).

These data thus demonstrate that Zeb1 also after birth facilitates CM cell cycle activity, but unlike before birth, favors CM endoreplication and not division leading to increased CM ploidy.

## Discussion

While most studies focus on regeneration of CMs at either fetal or adult stages, the understanding of the transcriptional changes leading to the transition from a fetal to a postnatal CM phenotype is relatively poor [49]. The postnatal period for a CM is characterized by a change from hyperplastic to hypertrophic growth [38], a switch from glycolytic to fatty acid oxidation [50], and sarcomere maturation with adult contractile isoforms [14, 73]. Together, this leads to cell cycle exit and terminal CM differentiation [49]. Through a systematic, high-resolution scRNA-seq approach, analyzing mouse CMs around the time of birth, we identified distinct transcriptional profiles of CM subpopulations, and identified multiple master TFs that control numerous cell cycling genes in CMs. Specifically, detailed analysis of ZEB1, a previously unknown factor in CM cell cycling, showed that before birth, ZEB1 works as a key regulator of cell cycle promoting genes and is required for CM proliferation. Yet, after birth ZEB1 mediated CM cell cycling occurs through endoreplication and leads to polyploid CMs. In agreement with the literature [11, 32, 61], the number of dividing CMs is indeed also very scarce after birth in our data, and it seems likely that CMs also in the mouse already initiates the final round of cell cycling before birth at which point ZEB1 seems to impact CMs. This would be similar to what is observed in humans, where CMs starts the process of terminal differentiation with increased polyploidy already before birth [18]. Thus, it is possible that ZEB1 represents a mechanism where CM proliferation is sustained by ZEB1 but at some point, mitotic stress is reached forcing the self-limiting DNA damage response to initiate terminal differentiation and avoid cancer. By this, ZEB1 may ensure polyploidy to achieve high production of RNA and proteins required for the high muscle work of fully differentiated CMs in adulthood, and its timed downregulation eventually caused by a self-limiting DNA response then enables p21 mediated CM differentiation. This could be in line with the emerging theory that polyploidy is an essential biological mechanism for tissue differentiation and homeostasis [19].

Thus, our novel approach has allowed us to acquire valuable new biological information that may be used further to understand the underlying mechanisms of the switch from CM proliferation to polyploidy occurring around the time of birth in mammals. Whether Zeb1 re-expression also underly the cell cycle activity observed in discrete CMs after MI remains to be determined but could provide a target to enable CM proliferation or forced CM polyploidy at this stage to increase CM mass and compensating the CM loss after MI.

Several scRNA-seq studies on in vivo CMs have recently provided new knowledge on heart development by identifying genes that are differentially expressed at different stages of development. Yet, many of the studies are limited in the number of CMs detected [5, 10, 30, 40] restricting subsequent detailed analysis such as TF binding site enrichment studies. Other studies fail to implement data on ploidy and cell cycle status [23, 39]. To our knowledge analysis of TFs in CMs based on scRNA-seq has been described in only four settings for CM development, but none used ploidy stratification as performed herein [10, 27, 30, 39]. However, in a recent study, Yekelchyk et al. showed transcriptional homogeneity by scRNA-seq of adult rod-shaped mono- and multi-nucleated ventricular CMs, although, not performing TF analysis [77]. With the established protocol, we unravel the uniqueness of in vivo cycling CMs in the G2/M phases around the time of terminal CM differentiation, and besides Zeb1 identified several TFs potentially involved in the process of G2/M phases completion. *Mycn* and *Myc* were shown to enhance not only S-phase progression, but also G2/M completion, in agreement with recent data [8, 60]. The more novel players in CM cell cycling: *Arnt*, *Zeb1*, *Sp1*, and *Egr1* specifically promoted S-phase progression herein with high efficiency, yet all four seemed to leave the postnatal CMs in a polyploid state thus favoring karyo-/cytokinesis failure. Whereas *Arnt* has been associated with hypoxia [74], *Egr1* seems to be implicated in several pathologies of the cardiovascular system [31], and *Sp1* is a well-known TF in cell growth and peripherally related to CM cell cycling [21]. *Zeb1* is mainly described for its enhancing role in epithelial to mesenchymal transition during cancer and embryonic development [81], but has recently been linked also to Hematopoietic stem cell renewal and asymmetric cell division [1]. Furthermore, ZEB1 has been suggested to interact directly with the Hippo pathway in cancer cells through YAP [34]. The functional roles of the TFs, however, were only predicted bioinformatically and not directly examined besides EdU incorporation and determination of ploidy. Herein, we found *Zeb1* to regulate the highest number of genes related to CM cell cycling in our dataset. Knockdown of *Zeb1* in E16.5 CMs led to impaired S-phase progression with reduced expression of the major cell cycle regulators *Ccnd1*, *Ccnb1*, *Ccnd3*, and *Cdk1* as well as a decrease in the level of *Cenpe*, *Cenpf*, *Aurkb*, and *Aurka*, which are all genes expressed in the G2/M phases of the cell cycle, confirming our bioinformatic prediction of ZEB1 as a regulator of the cell cycle in CMs before birth. *Ccnb1*, *Ccnd1*, *Cdk1*, and *Cdk4* are known regulators of CM cell cycling and overexpression of these four factors was recently found to promote proliferation of post-mitotic CMs [46]. Manipulating *Zeb1* by overexpression in postnatal CMs showed that ZEB1 after birth maintains cell cycling in the form of endoreplication specifically governed by Cyclin E while likely inhibiting cell cycle exit through *Cdkn1a* (p21) regulation. It is known that p21 binds and inhibits CDK1/Cyclin B1 thereby blocking G1/S and G2/M phase transitions, and that p21 knockout increases CM ploidy significantly [66], while p21 expression forces CM cell cycle exit [65]. In agreement, S-phase progression and ploidy was high in ZEB1 expressing CMs. Since, p21 blocks endoreplication [66] through co-repressing Cyclin E [24], the major cyclin of endoreplication [82], it is thus intriguing to speculate that ZEB1 herein also downregulates p21 and hereby increases Cyclin E2 expression resulting in endoreplication of cycling CMs and polyploidy as observed for a fraction of CMs both in vitro and in vivo. In this regard, it is important to note, that Cyclin D1 in parallel was decreased upon ZEB1 overexpression. Whether this in turn inhibits G1/S phase transition and prevents non-cycling CMs from further entering the cell cycle remains elusive but could explain why only a proportion of CMs undergo S-phase progression despite expressing ZEB1. Yet, we did observe that all ZEB1 overexpressing CMs reduced their size in vitro, which therefore likely represents another mechanism. The smaller size of ZEB1 expressing CMs at early timepoints was accompanied by an increase in the major muscle size inhibitors *Mstn*, *Ccng2*, and *Tead1*, and downregulation of the Yap targets *Axl* and *Ctgf.* All these genes are related to cell size regulation, and recently ZEB1 was demonstrated to inhibit skeletal muscle cell size in mouse [59]. Thus, the reduced CM size and decrease in Myh6 fit very well with ZEB1 preventing cell cycle exit, while promoting S-phase progression in the early phase. At P8 in vivo, ZEB1-mediated endoreplication then results in polyploid CMs of increased size, which agrees with the literature [82]. Interestingly, our studies indicate that ZEB1 regulates a distinct set of genes before and after birth, thereby promoting CM proliferation before birth, while favoring polyploidization when reintroduced after birth. To our knowledge this clear molecular switch from proliferation to endoreplication around birth has not previously been shown for one TF. Thus, the decrease in Zeb1 expression occurring around birth may contribute to cell cycle arrest and terminal CM differentiation, as also observed for other TFs [17]. One example is YAP1, which as part of the Hippo pathway supports CM proliferation during embryonic development in combination with its interaction partner TEAD1 [43]. Thus, in the postnatal period downregulation of YAP1 and TEAD1 is required for CM cell cycle arrest [22]. Postnatal upregulation of YAP1 retain CM proliferative capacity after birth causing cardiomegaly and heart failure [49]. MYC, which was also detected in our TF analysis, induces CM proliferation during development, while expression of MYC in adult mice leads to an increase in polyploid cells [76]. However, in another study it was suggested that combinational overexpression of MYC and Cyclin T1 induce CM proliferation without any notable change in CM size and nucleation [8]. Thus, it is intriguing to speculate, whether overexpression of Zeb1 in combination with other genes or TFs also after birth could promote CM proliferation rather than endoreplication, and by this may be a target for therapeutic perspectives in MI patients.

In conclusion, we here provide new knowledge on Zeb1’s cell cycle promoting actions in CMs as well as a comprehensive scRNA-seq of CMs before and after birth, which may be used to further dissect the switch from a proliferative to a terminally differentiated high power beating CM.

### Supplementary Information

Below is the link to the electronic supplementary material.

Supplementary file1 (DOCX 13965 KB)

## Data Availability

ScRNA-seq data that support the findings of this study have been deposited in the Gene Expression Omnibus (GEO) under the accession code GSE162959. All other data supporting the findings of this study are available from the corresponding authors on reasonable request.

## Code availability

All codes central for the conclusions made in this manuscript are available from the corresponding author on reasonable request.
