# Supplementary material for: Ploidy-stratified single cardiomyocyte transcriptomics map Zinc Finger E-Box Binding Homeobox 1 to underly cardiomyocyte proliferation before birth
Source: Basic Res Cardiol. 2023 Mar 2;118(1):8. doi: 10.1007/s00395-023-00979-2 (PMC9981540; doi:10.1007/s00395-023-00979-2)
Supplement: Supplementary file 1 — Supplementary file1 (DOCX 13965 KB) [file 395_2023_979_MOESM1_ESM.docx]

**Ploidy-stratified single cardiomyocyte transcriptomics map Zinc Finger E-Box Binding Homeobox 1 to underly terminal cardiomyocyte differentiation.**

**Basic Research in Cardiology**

^1,2 #^Sara Thornby Bak, ^1,2 #^Eva Bang Harvald, ^1,2 #^Ditte Gry Ellman, ^1,2^Sabrina Bech Mathiesen, ^1,2^Ting Chen, ^1,2^Shu Fang, ^1,2^Kristian Skriver Andersen, ^3^Christina Dühring Fenger, ^2,4^Mark Burton, ^2,4^Mads Thomassen, and ^1,2 *^Ditte Caroline Andersen.

^1^Andersen group, Dep. of Clinical Biochemistry, Odense University Hospital, Denmark.

^2^Clinical Institute, University of Southern Denmark, Odense, Denmark.

^3^Amplexa Genetics, Odense, Denmark.

^4^Department of Clinical Genetics, Odense University Hospital, Denmark

^#^S.T.B, E.B.H. and D.G.E. contributed equally to this work.

^*^Corresponding author: Ditte C. Andersen; E-mail, [dandersen@health.sdu.dk](file:///Users/dandersen/Downloads/dandersen@health.sdu.dk)

**
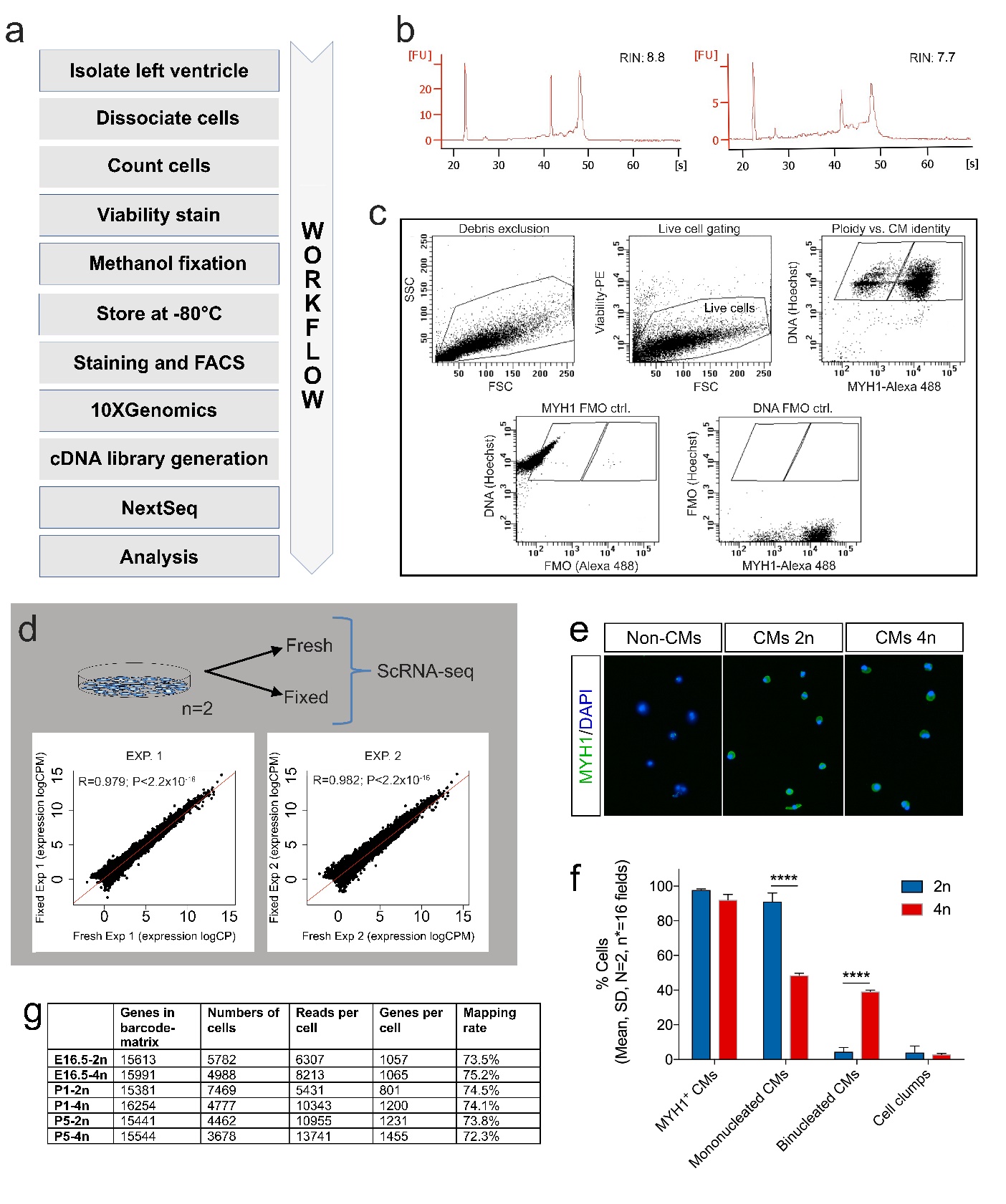
**

**Supplementary Fig. 1 Overview of new scRNA-seq approach a**, Schematic presentation of the study workflow until scRNA-seq data analysis. **b**, Electropherograms displaying the RNA integrity number (RIN) of dissociated E16.5 mouse cardiac cells before and after FACS. **c**, FACS sorting strategy used including FMO controls. **d**, Correlation of scRNA-seq data for live and fixed myogenic cells. **e, f**, Quantification of CM marker identity (MYH1), ploidy (DAPI), and cell clumping in FACS sorted fractions (NMs, 2n- and 4n CMs) based on immunofluorescence (Fisher’s LSD test, **** P ≤ 0.0001). **g**, Raw data from scRNA-seq of FACS-sorted diploid (2n) and tetraploid (4n) mouse heart cells (E16.5, P1 and P5).


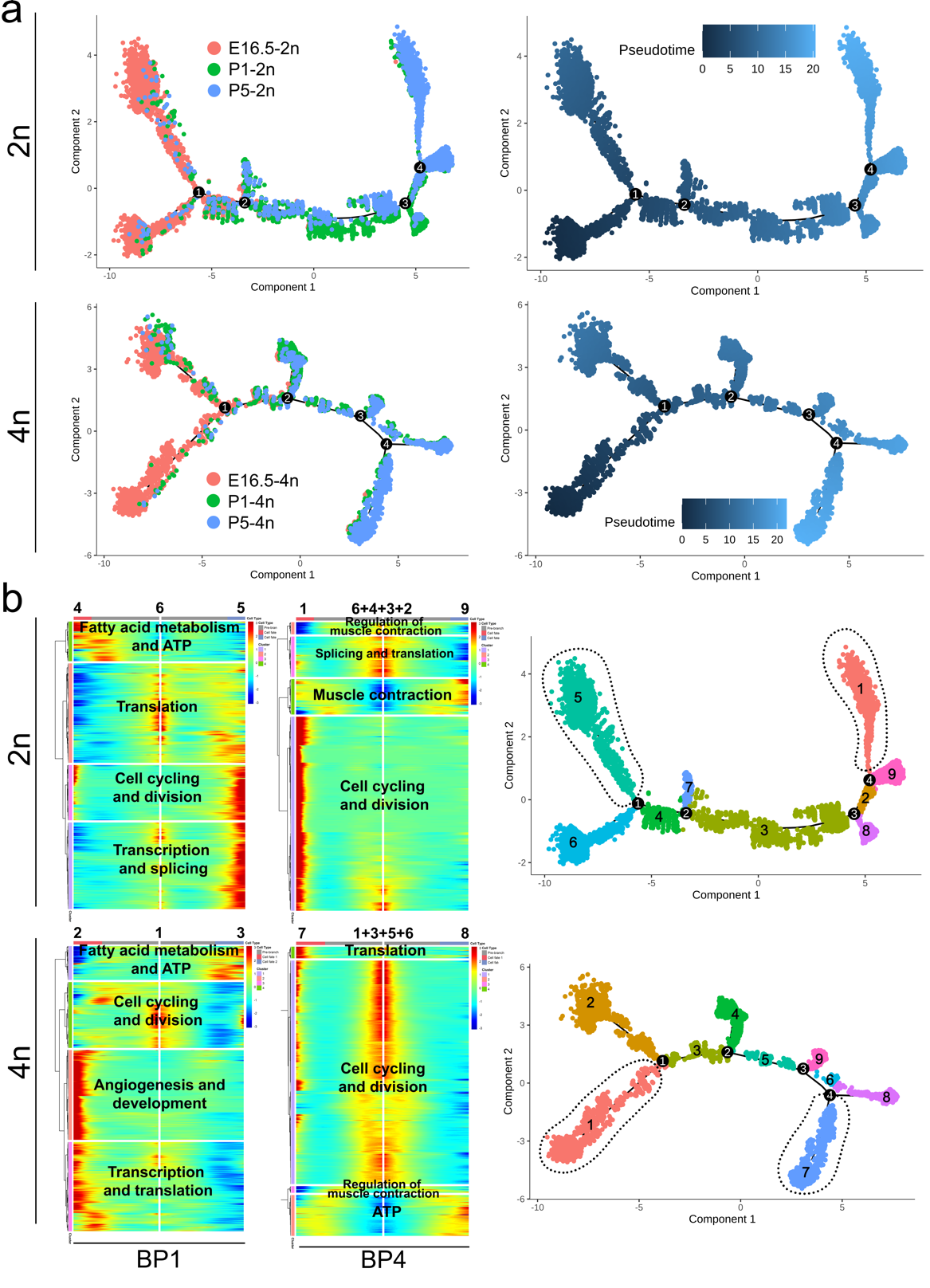


**Supplementary Fig. 2 CM- cell cycle activity through pseudotime. a, b**, Trajectory analysis of 2n- and 4n CMs at different developmental stages. Original cell identity and pseudotime (**a**), and trajectory state including three branchpoints (BP) (**b**) are shown. Based on BP analysis, CMs encircled by a dotted line (**b**; right) represent bifurcations with a higher cell cycle activity based on gene expression patterns.


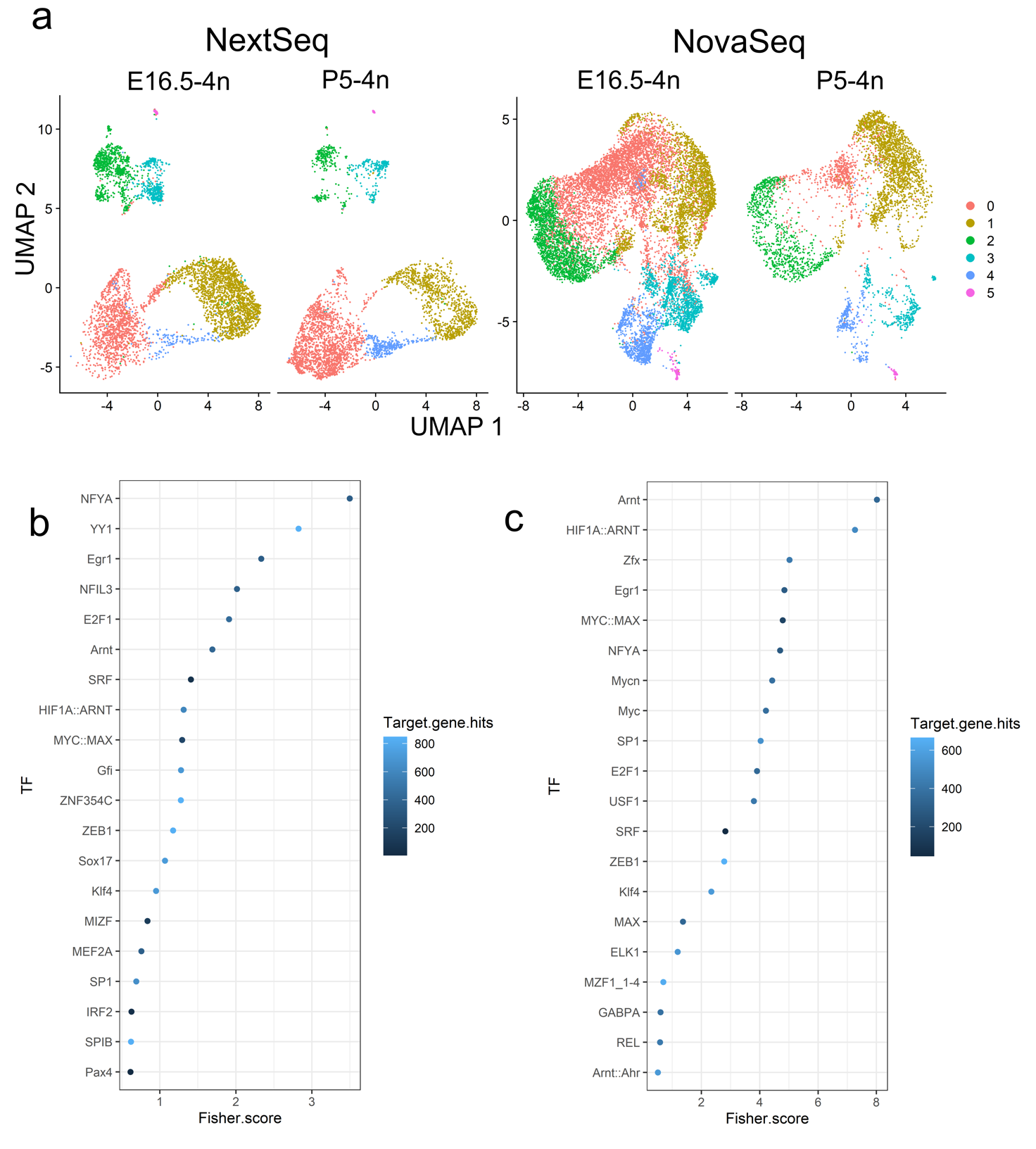


**Supplementary Fig. 3 Comparative TF analysis of NextSeq and NovaSeq generated data. a**, UMAP clustering of E16.5-4n and P5-4n CMs using NextSeq and NovaSeq generated data, respectively. **b,** Enriched TFs (oPOSSUM <http://opossum.cisreg.ca/oPOSSUM3/>) corresponding to the higher expressed genes in 4n-G2/M-E16.5 cluster versus the 4n-G2/M-P5 cells in a direct comparison between NextSeq and NovaSeq generated data, respectively.


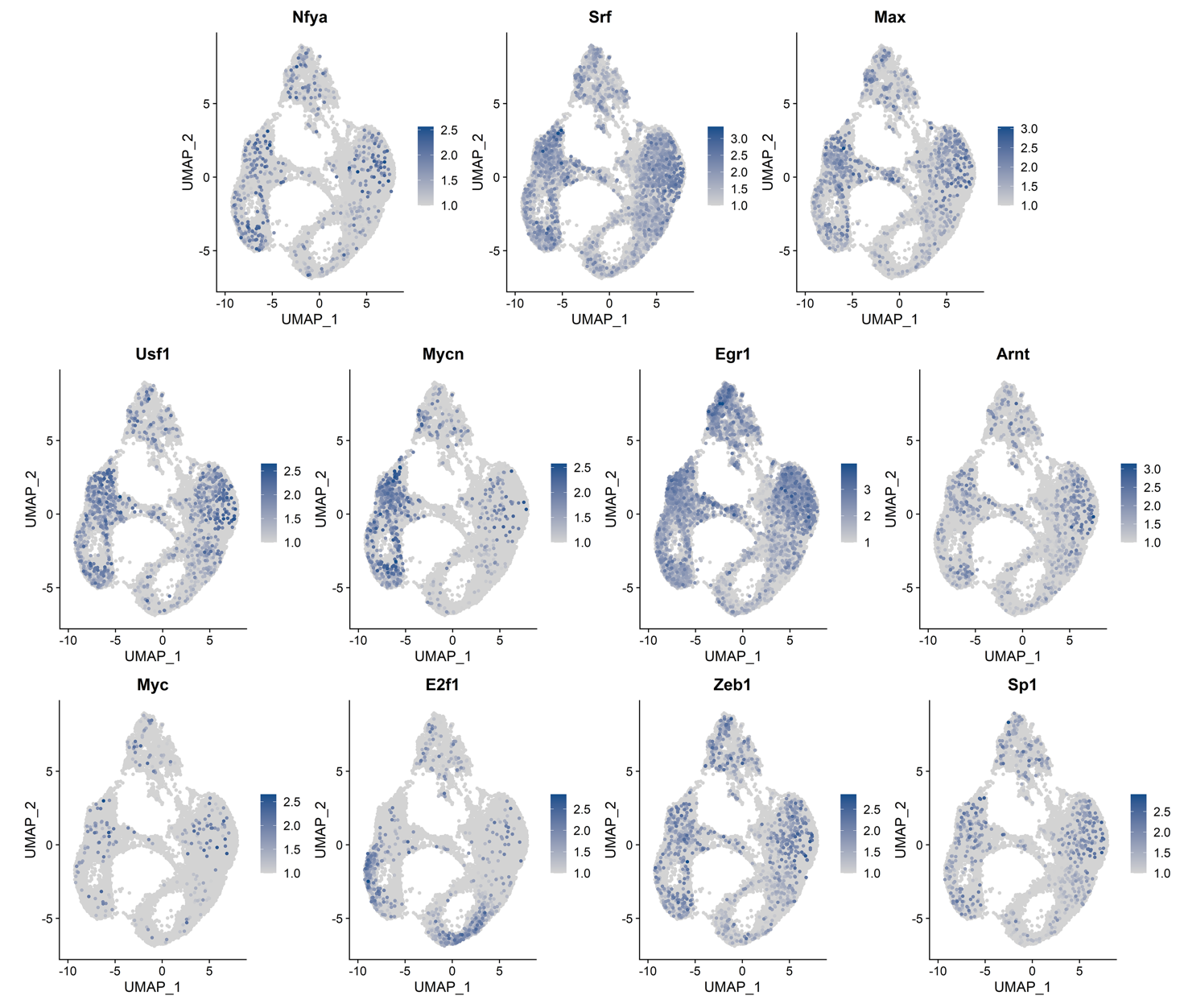


**Supplementary Fig. 4 Expression in CMs of selected transcription factors (TFs) in clusters of cells.** UMAP plots for selected TFs across developmental stages and ploidy of CMs.


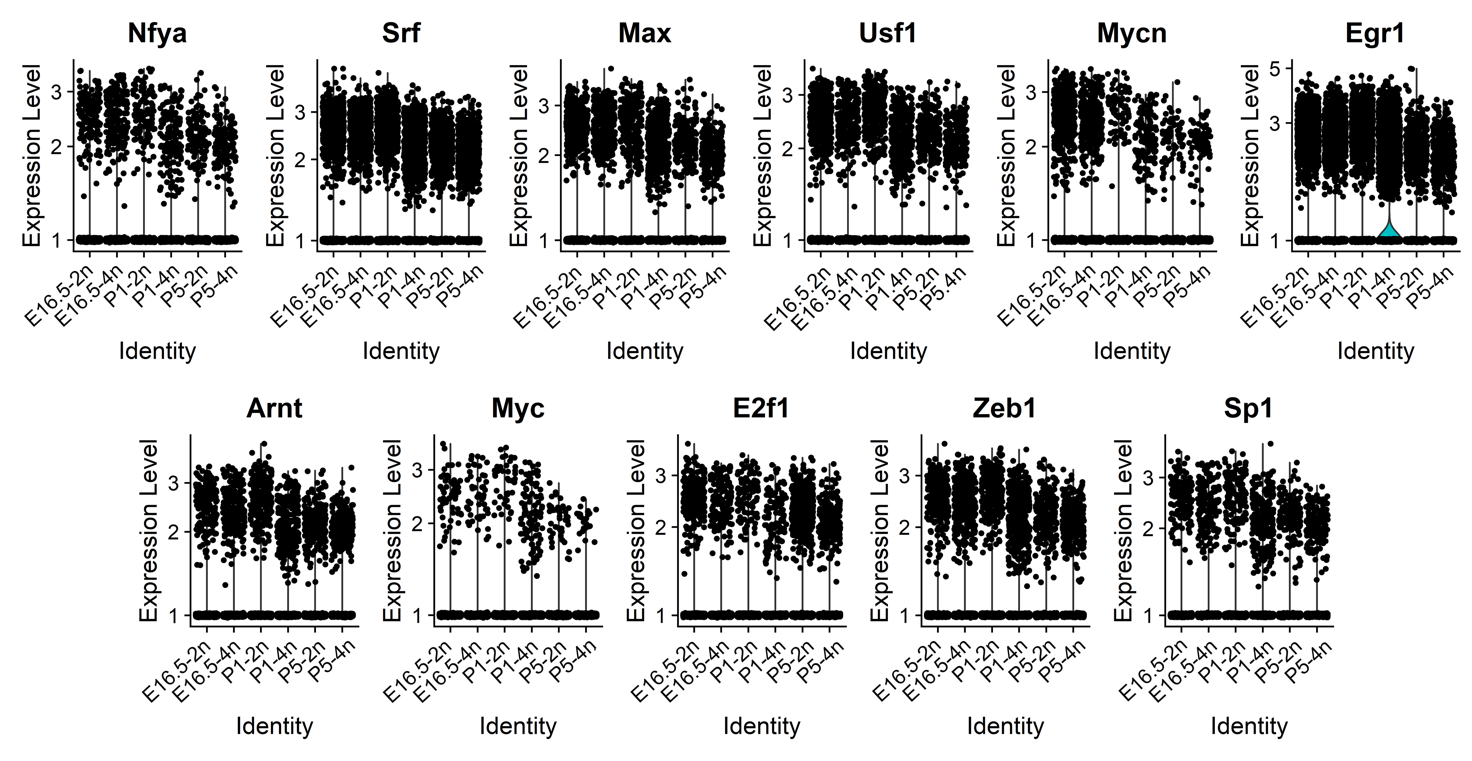


**Supplementary Fig. 5 Expression in CMs of selected transcription factors (TFs) over time during development.** Violin plots illustrating TF expression levels in CMs at each development stage and ploidy to emphasize expression dynamics.


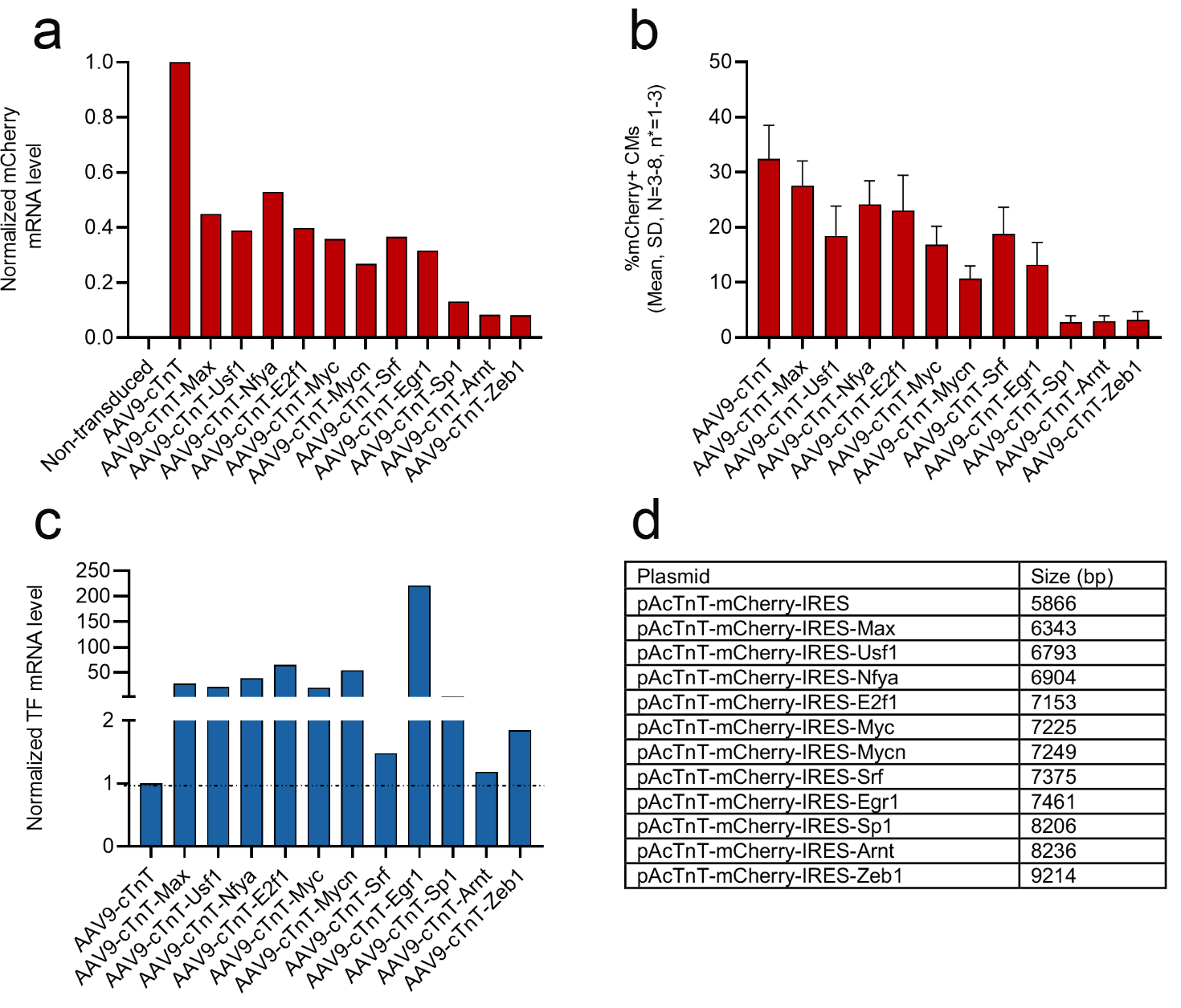


**Supplementary Fig. 6 Efficiency of AAV9 CM transduction *in vitro*. a, b** mRNA levels of mCherry (**a**) and TFs (**b**) were quantified by qRT-PCR including normalization against stably expressed endogenous controls (*B2m* and *Gapdh*) after 72h of AAV9-cTnT-TF CM AAV9-cTnT-TF transduction. **c,** Percentages of mCherry^+^ CMs were assessed by flow cytometry 72h after AAV9-cTnT-TF transduction. **d,** Overview of the size (base pairs, bp) of the plasmid constructs for the generated AAV9s.


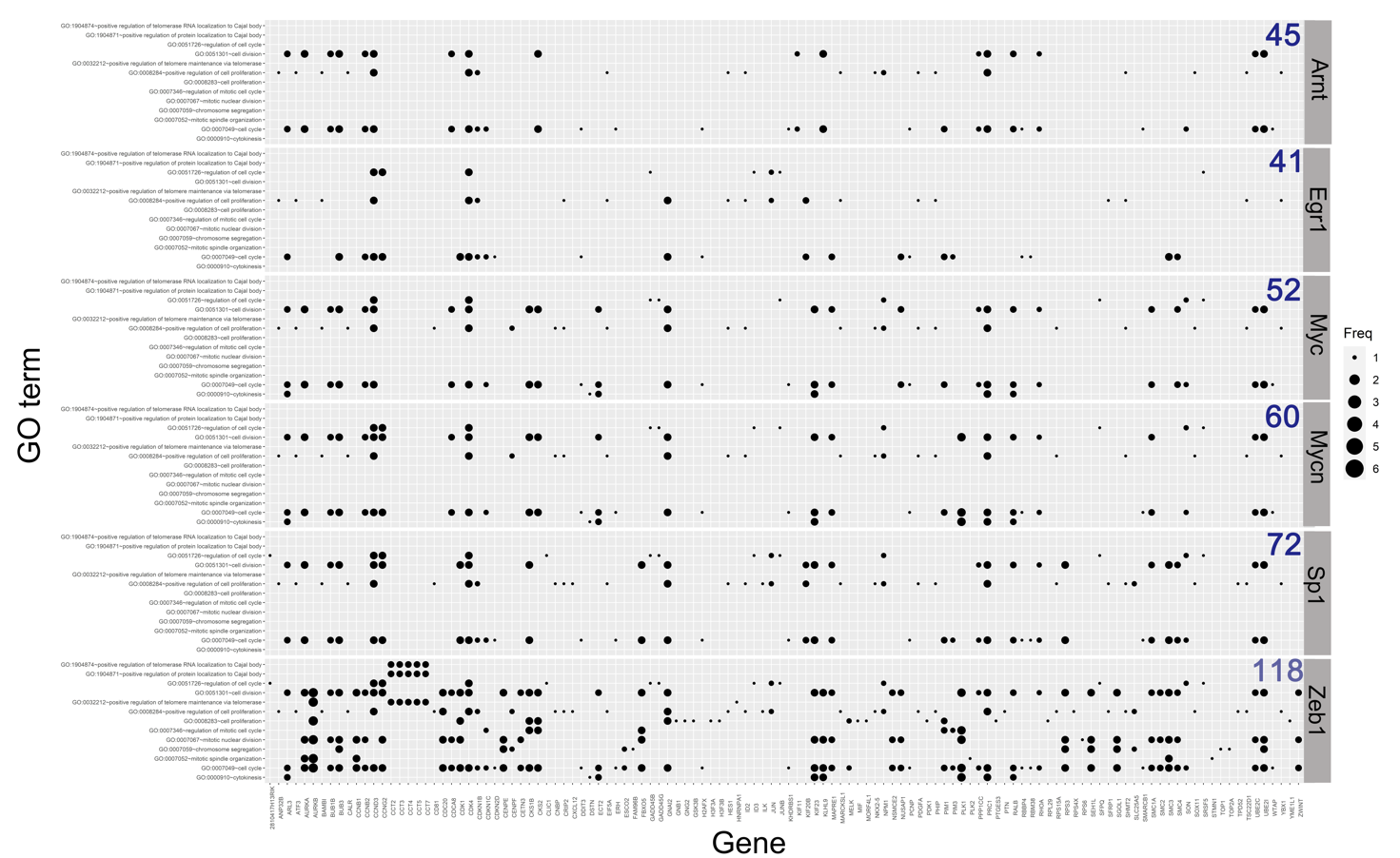


**Supplementary Fig. 7 Cell cycle related genes in CMs regulated by scRNA-seq identified TFs.** The number of cell cycle related genes affected is designated for each TF (Blue), whereas the size of the dot indicates the frequency of a gene across the cell cycle related GO terms indicated.


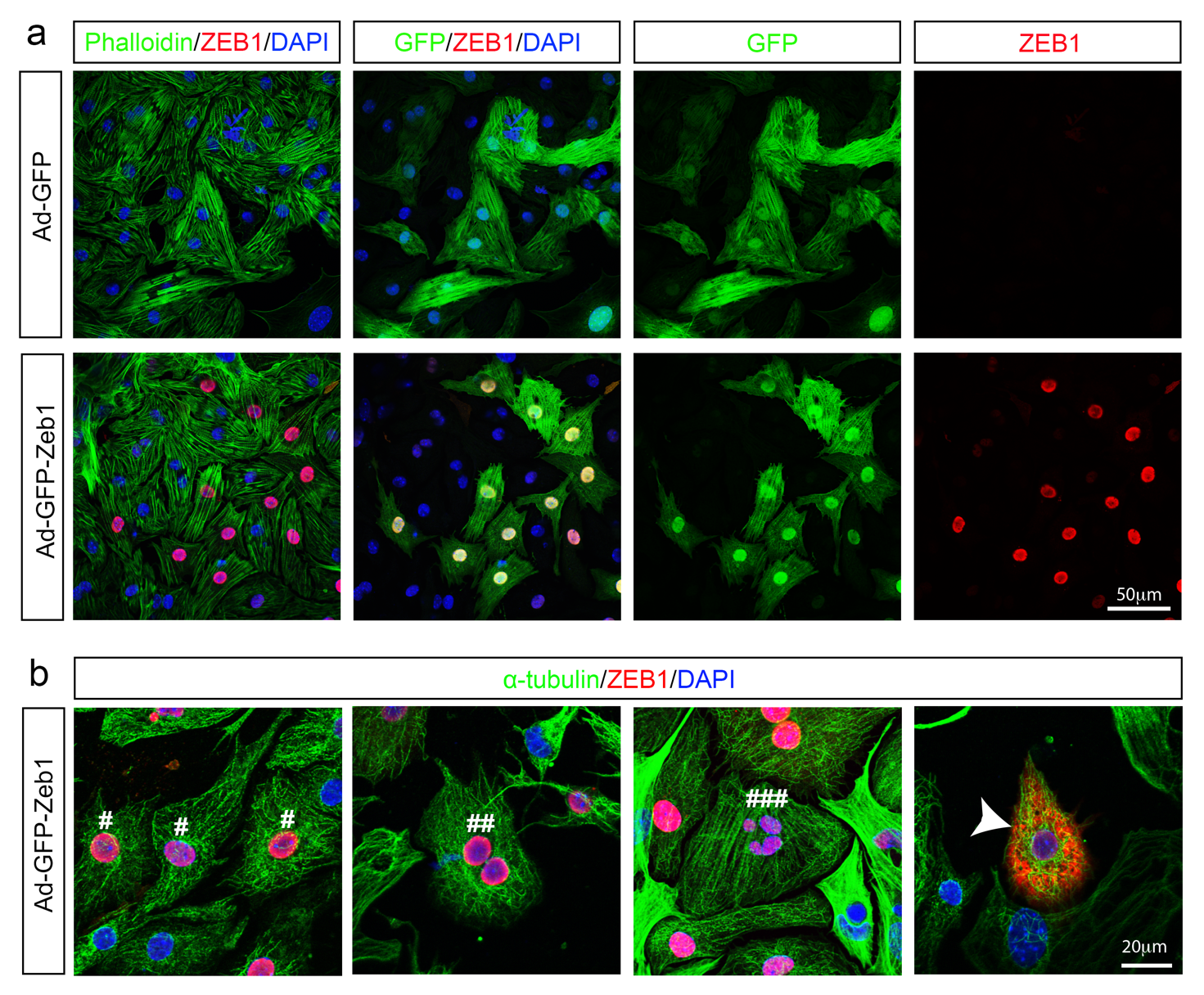


**Supplementary Fig. 8 ZEB1 localization in adenovirus transduced CMs.** **a,** Confocal images of Ad-GFP (top panel) or Ad-GFP-Zeb1 (lower panel) transduced CMs after 72h of transduction (phalloidin (F-actin) or GFP, green; ZEB1, red; DAPI (cell nuclei), blue). **b,** Confocal images of Ad-GFP-Zeb1 transduced CMs after 72h of transduction (α-tubulin, green; ZEB1, red; DAPI (cell nuclei), blue) Mononucleated CMs (#), binucleated CM (##), tetranucleated CM (###), cytoplasm localized ZEB1 (arrowheads).

**Supplementary Table 1: Plasmids used for generation of AAV9**

| Plasmids: Mouse tagged ORF clones from Origene | |
| --- | --- |
| Sp1 | MR225692 |
| Srf | MR208120 |
| Mycn | MR207382 |
| Arnt | MR210673 |
| Max | MR223868 |
| Usf1 | MR224006 |
| Egr1 | MR227136 |
| Myc | MR227353 |
| E2f1 | MR206856 |
| Zeb1 | MR223095 |
| Plasmid: Costume made by Thermo Fischer GeneArt | |
| Nfya |  |

**Supplementary Table 2: Mouse qPCR primers -5’ to 3’**

| **Name** | **Forward** | **Reverse** | **Sample volume (ng)** | **Annealing temp (°C)** |
| --- | --- | --- | --- | --- |
| *Arnt* | GGCTCCTACAAGCCATCTTT | AGCTGCTCTCGAAGTTTATCC | 2 | 60 |
| *AurkA* | CCTCAGGCTGTATGGCTATTT | GCAATCTTCAACTCTCCGTTTG | 2 | 60 |
| *AurkB* | CTTCGCCGAGAGATCGAAAT | CTTCTTCTTGTGGCAGTAGGT | 2 | 60 |
| *Axl* | CAAGGGATCGTCTCACATCTTC | CATCTCGGTCCAAAGACTCATC | 2 | 57 |
| *B2m* | ATGGCTCGCTCGGTGACCCT | TTCTCCGGTGGGTGGCGTGA | 2 | 57 |
| *Ccnb1* | GGTGTAACGGCCATGTTTATTG | GGAGAGCTCCATGAGGTATTTG | 2 | 60 |
| *Ccnd1* | GAAGTGGAGACCATCCGCCGC | CCTCACAGACCTCCAGCATCCAGG | 2 | 57 |
| *Ccnd3* | TGTGGCTCCATGGCAGTTGCG | AAGTCGTGCGCAATCACGGCA | 2 | 57 |
| *Ccne2* | TGGCCACCTGTACTGTCTGGAGG | ACCTCCTGTGAACATGCCCAGCTTA | 2 | 57 |
| *Ccng2* | CGACACGATGAAGGATTTGGGGGC | CTCCGGGGTAGCCTCCATCAAGATC | 2 | 57 |
| *Cdk1* | GTACCGATCTCCAGAAGTGTTG | CCACTTGGGAAAGGTGTTCT | 2 | 60 |
| *Cdk4* | CTGAGGACATACCTGGACAAAG | GCCATCTGGTAGCTGTAGATTC | 2 | 60 |
| *Cdkn1a* | ATCCAGACATTCAGAGCCACAGGCA | GCATCGCAATCACGGCGCAA | 2 | 60 |
| *Cdkn1b* | AGTCAGCGCAAGTGGAATTT | AGTAGAACTCGGGCAAGCTG | 2 | 60 |
| *CenpE* | ACACACGATGATGGGTTCTG | CCTATTGGTGTCTTCCCGAATTA | 2 | 60 |
| *CenpF* | CAGAAGGTTGAAGACGGAAAGA | TACACCGCTTAAGTTCCTGTTC | 2 | 60 |
| *Ctgf* | ACTATGATGCGAGCCAACTG | CTCCAGTCTGCAGAAGGTATTG | 2 | 60 |
| *E2f1* | GATTCTGACGTGCTGCTCTT | AGCGTTTGGTGGTCAGATTTA | 2 | 60 |
| *Egr1* | CTTTCCTACTCCCAACACTGAC | TGAGTGGCGAAGGCTTTAAT | 2 | 60 |
| *Gmnn* | ACGGATGCTAGGCCGTGTAC | GCACCGTGTAGTTAGTTTACCAAGAG | 2 | 60 |
| *Max* | AACGTAGGGACCACATCAAAG | GTTGGTGTAGAGGCTGTTGT | 2 | 60 |
| *mCherry* | GACGGCGAGTTCATCTACAA | GGAGGTGATGTCCAACTTGAT | 2 | 60 |
| *Myc* | CTGCGACGAGGAAGAGAATTT | CCAAGTAACTCGGTCATCATCTC | 2 | 60 |
| *Mycn* | GGAGGACATCTGGAAGAAGTTT | CCTGAAGGATGACCGGATTAG | 2 | 60 |
| *Myh6* | CTACGCCTTCGTCTCTCAGG | CTTCTGTGCCATCAGGCTCT | 2 | 57 |
| *Mstn* | CAGCCTGAATCCAACTTAGG | TCGCAGTCAAGCCCAAAGTC | 4 | 60 |
| *Nfya* | CGTTCAGACAGGAGCCAATAC | CCTCTTAAGGATGCGGTGATAC | 2 | 60 |
| *Rpl_13A* | CGAAGATGGCGGAGGGGCAG | ACCACCACCTTCCGGCCCAG | 2 | 57 |
| *Sp1* | ACCAGGTGCAAACCAACAGA | AGAGCTGGGAGTCAGGGTAG | 2 | 60 |
| *Srf* | GTGAGACAGGCCATGTGTAT | TGACTTGCATGGTGGTAGAG | 2 | 60 |
| *Tead1* | AGGGCTTCTCTTCCATCTTTG | CTGACTCTGTCCATGCATTCT | 2 | 60 |
| *Usf1* | AACGGAGGGCTCAACATAAC | TCCAGCTGCAACTGATCTAAC | 2 | 60 |
| *Zeb1* | CTTACGGATTCACAGTGGAGAG | GTGAGCTATAGGAGCCAGAATG | 2 | 60 |
